# MED15 is upregulated by HIF-2α and promotes proliferation and metastasis in clear cell renal cell carcinoma via activation of SREBP-dependent fatty acid synthesis

**DOI:** 10.1038/s41420-024-01944-1

**Published:** 2024-04-22

**Authors:** Xiaoliang Hua, Shengdong Ge, Li Zhang, Qing Jiang, Juan Chen, Haibing Xiao, Chaozhao Liang

**Affiliations:** 1 Department of Urology, The Second Affiliated Hospital of Chongqing Medical University, Chongqing, China; 2 Department of Urology, The First Affiliated Hospital of Anhui Medical University, Hefei, China; 3 The Ministry of Education Key Laboratory of Laboratory Medical Diagnostics, the College of Laboratory Medicine, Chongqing Medical University, 400016 Chongqing, China

**Keywords:** Renal cell carcinoma, Cancer metabolism

## Abstract

Emerging evidence has highlighted that dysregulation of lipid metabolism in clear cell renal cell carcinoma (ccRCC) is associated with tumor development and progression. HIF-2α plays an oncogenic role in ccRCC and is involved in abnormal lipid accumulation. However, the underlying mechanisms between these two phenomena remain unknown. Here, MED15 was demonstrated to be a dominant factor for HIF-2α-dependent lipid accumulation and tumor progression. HIF-2α promoted MED15 transcriptional activation by directly binding the MED15 promoter region, and MED15 overexpression significantly alleviated the lipid deposition inhibition and malignant tumor behavior phenotypes induced by HIF-2α knockdown. MED15 was upregulated in ccRCC and predicted poor prognosis. MED15 promoted lipid deposition and tumor progression in ccRCC. Mechanistic investigations demonstrated that MED15 acts as SREBP coactivator directly interacting with SREBPs to promote SREBP-dependent lipid biosynthesis enzyme expression, and promotes SREBP1 and SREBP2 activation through the PLK1/AKT axis. Overall, we describe a molecular regulatory network that links MED15 to lipid metabolism induced by the SREBP pathway and the classic HIF-2α pathway in ccRCC. Efforts to target MED15 or inhibit MED15 binding to SREBPs as a novel therapeutic strategy for ccRCC may be warranted.

## Introduction

Renal cell carcinoma (RCC) is one of the most frequently diagnosed malignancies worldwide, and clear cell renal cell carcinoma (ccRCC) is the most common histologic subtype, accounting for approximately 75% of RCCs [1]. ccRCC is characterized by malignant epithelial cells with a clear cytoplasm owing to the accumulation of large amounts of lipids and glycogen that are removed in histological staining [2]. Biallelic inactivation of von Hippel–Lindau (VHL) is the typical molecular feature of ccRCC patients, with approximately 85% of patients showing this alteration [3]. Loss of pVHL function can affect various processes; stabilization and accumulation of hypoxia inducible factor (HIF) are the best-known functions of pVHL, and loss of these functions contributes to a neoplastic phenotype [4]. Of the HIF subunits, HIF-1α and HIF-2α have functionally distinct roles [5]. Recent evidence seems to suggest that HIF-2α has a more oncogenic role, is necessary and sufficient to promote tumor growth and can activate protumorigenic target genes [6]. Moreover, HIF-2α has been validated as a therapeutic target in ccRCC [7], and a HIF-2α antagonist was found to inhibit tumor progression in ccRCC xenograft tumor models [8]. A phase II clinical trial was conducted to evaluate the safety and efficacy of a HIF-2α antagonist in patients with advanced ccRCC [9]. However, given the rapid increase in resistance to HIF-2α antagonists, other strategies to target HIF-2α-expressing ccRCC cells need to be explored [7, 10]. Therefore, systematically exploring potential HIF-2α target genes and their functions in ccRCC may provide a more reliable theoretical basis for treatment.

Abnormal lipid deposition is another feature of ccRCC, and the levels of fatty acids, triglycerides, and cholesterol are higher in ccRCC tissues than in normal kidney tissues [11]. Elevated lipid accumulation can sustain endoplasmic reticulum homeostasis to promote tumor growth [12]. In recent years, HIF-2α has been shown to be involved in abnormal lipid accumulation [12, 13]. PLIN2-dependent lipid accumulation in ccRCC is modulated by HIF-2α [12]. Downregulation of the CPT1A gene can lead to a defective lipid transport system to promote lipid deposition and tumor growth, and CPT1A has been shown to be a direct target gene of HIF-2α [13]. Although abnormal lipid deposition and HIF-2α accumulation are known to be dysregulated in ccRCC, the underlying mechanisms between these two phenomena remain to be explored.

In eukaryotes, the initiation of gene transcription requires RNA polymerase II, general transcription factors (TFIIA, TFIIB, TFIID, TFIIE, TFIIF, and TFIIH), DNA-binding transcription factors and transcription coactivators. The mediator complex is generally considered a transcription coactivator that forms the bridge between gene-specific transcription factors and the RNA polymerase II machinery [14, 15]. Moreover, different transcription factors interact with only one subunit, or in some cases a few subunits, of the mediator complex, leading to gene-specific physiological effects [16]. MED15 is a subunit of the mediator complex and functions as a transcription cofactor. Recent studies have shown that MED15 plays a vital role in controlling lipid homeostasis [17]. Sterol regulatory element binding protein (SREBP) family members are critical regulators of cholesterol and fatty acid homeostasis, and MED15 can interact directly with SREBPs to activate SREBP transcriptional activity and promote SREBP target gene expression [18, 19]. Moreover, the mediator complex displays context-dependent functions. Whether MED15 plays a role in lipid accumulation, tumor cell proliferation, and cell viability in ccRCC and its possible regulatory mechanism remain unclear.

In the present study, we found that MED15 induced lipid accumulation, cell proliferation, and tumor metastasis via transcriptional activation of SREBPs in ccRCC cells. MED15 also activated the PLK1/AKT pathway to further promote the transcriptional activation of SREBPs. The activation of SREBPs by MED15 was partially alleviated by knockdown of PLK1. MED15 was shown to be a direct target gene of HIF-2α and participated in the lipid accumulation induced by HIF-2α in ccRCC. In addition, MED15 knockdown reduced lipid accumulation and suppressed tumor growth in vivo. Analyses of clinical samples of ccRCC confirmed the high expression and activity of MED15 in ccRCC and the negative correlation of MED15 expression with patient prognosis. Together, these findings provide new insight into lipid accumulation in ccRCC and suggest that targeting MED15 may be a new strategy for therapeutic intervention in ccRCC.

## Results

### MED15 was upregulated and predicted poor prognosis in ccRCC

**Fig. 1:**
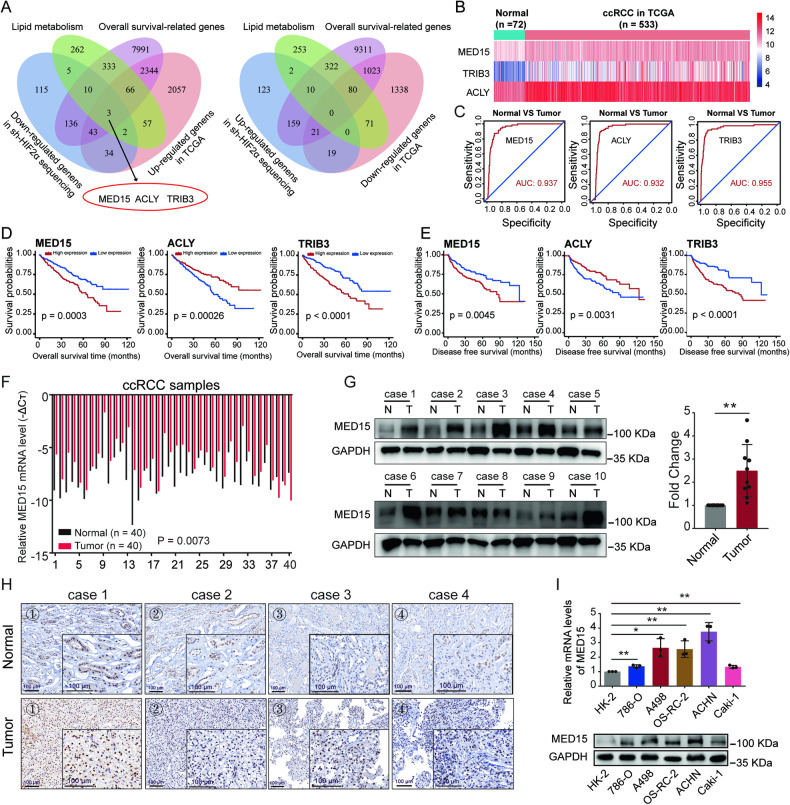
MED15 was upregulated in ccRCC. **A** Venn diagram of the results for four independent datasets, including lipid metabolism-related genes, survival-related genes and differentially expressed genes between normal and tumor samples from the TCGA dataset, and differentially expressed genes from transcriptome sequencing data obtained after HIF-2α knockdown. Three genes (MED15, ACLY, and TRIB3) were identified. **B** Heatmap showing the expression of MED15, ACLY, and TRIB3 in normal and tumor samples from the TCGA dataset. **C** The receiver operating characteristic (ROC) curves for MED15, ACLY, and TRIB3. Kaplan–Meier survival curves showed lower overall survival **D** and disease-free survival (DFS) times **E** for the high MED15, ACLY, and TRIB3 expression groups. **F** The mRNA levels of MED15 were increased in 40 ccRCC samples compared with 40 paired normal renal samples. **G** The protein levels of MED15 were increased in ccRCC samples compared with matched normal renal samples, as detected by western blot assay. **H** Immunohistochemical staining for MED15 in ccRCC samples and matched normal renal samples (scale bar: 100 μm). **I** The mRNA and protein levels of MED15 in five ccRCC cell lines and the HK-2 cell line (n = 3). The results are expressed as the mean ± standard deviation (SD). * indicates *P* < 0.05; ** indicates *P* < 0.01.

ccRCC is characterized by abnormal accumulation of lipids [11]. HIF-2α functions as an oncogene to promote tumor growth and metabolic reprogramming [12, 13]. To disclose the underlying association between these two phenomena, differentially expressed genes from the transcriptome sequencing results after HIF-2α knockdown, lipid metabolism-related genes, survival-related genes and differentially expressed genes between normal and tumor samples in TCGA dataset; three genes were selected for further analysis: MED15, ACLY, and TRIB3 (Fig. 1A). The overall expression levels of the three genes in the TCGA-KIRC dataset are shown in Fig. 1B; all three genes showed high expression in ccRCC tissues versus normal tissues. The clinical diagnostic value of these genes was evaluated using receiver operating characteristic (ROC) curves, and high area under the curve (AUC) values were found for these three genes, suggesting good ability to distinguish ccRCC from normal tissues (Fig. 1C). Patients were divided into high (above the 50^th^ percentile) and low (below the 50^th^ percentile) expression groups based on gene expression in the TCGA dataset. Kaplan–Meier survival curves suggested that patients in the high MED15, ACLY, and TRIB3 expression groups had lower overall survival and disease-free survival (DFS) times than those in the low MED15, ACLY, and TRIB3 expression groups (Fig. 1D, E). A recent study showed that TRIB3 mediates ccRCC progression and that the expression of TRIB3 is increased in response to hypoxia, which is directly regulated by HIF-1α [20]. ACLY is involved in carcinogenesis and lipid accumulation in ccRCC and is regulated by the VHL/PPARγ axis, independent of HIF-1α [21]. MED15 knockdown inhibited the proliferation and migration of ccRCC cells in vitro [22]. However, the roles of MED15 in lipid metabolism in ccRCC and the specific mechanism remain unclear. After a comprehensive evaluation of these results, we selected MED15 for subsequent experiments.

To further clarify the expression and clinical significance of MED15 in ccRCC, additional bioinformatics analyses were conducted. MED15 mRNA levels were significantly higher in ccRCC tissues than in normal renal tissues (Fig. S1A), and higher MED15 levels were found in ccRCC patients with higher stage, higher grade, higher T stage, N1 stage, and M1 stage (Fig. S1B, C). Furthermore, patients with poor prognosis had higher MED15 expression than patients with good prognosis (Fig. S1C). Higher MED15 expression in ccRCC tissues versus normal tissues was further confirmed in four datasets derived from the GEO database (Fig. S1D). Assessment of the relationship between MED15 mRNA expression and clinicopathological parameters showed that patients with high MED15 expression tended to have advanced stage and grade and poor prognosis (Table S1). Univariate and multivariate analyses showed that MED15 is an independent prognostic marker for overall survival (Table S2) and DFS (Table S3) in ccRCC patients. To further confirm these results, we examined the expression levels of MED15 in ccRCC patients using quantitative reverse transcription-polymerase chain reaction (qRT‐PCR) (Fig. 1F), western blotting (Fig. 1G), and immunohistochemistry (IHC) (Fig. 1H). We found high mRNA and protein levels of MED15 in ccRCC tissues versus normal tissues. Moreover, similar results were found in ccRCC cell lines (Fig. 1I). Taken together, these results suggest that MED15 expression is increased in ccRCC and that MED15 may act as an oncogene.

### MED15 promoted the progression of ccRCC

**Fig. 2:**
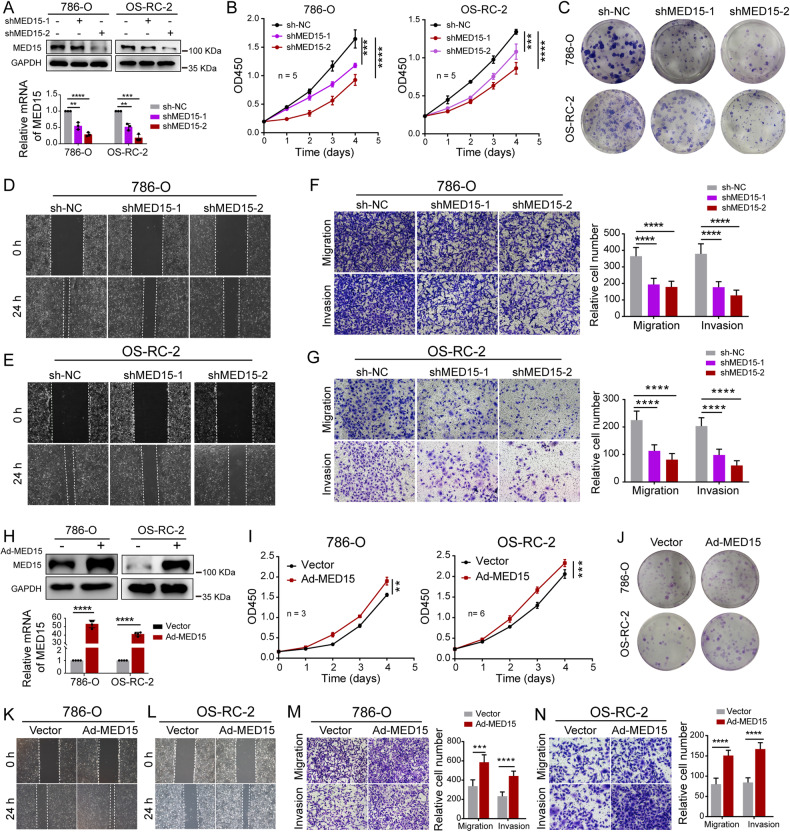
MED15 promoted the progression of ccRCC. **A** Western blot assay and qRT–PCR revealed that MED15 protein and mRNA levels were efficiently knocked down in RCC cells with MED15 knockdown. **B** Growth curves of RCC cells with MED15 knockdown detected by CCK-8 assay (n = 5). **C** The colony formation assay showed a decreased proliferation rate of RCC cells with MED15 knockdown at the second week of cultivation. **D**, **E** The wound healing assay indicated decreased migration of RCC cells with MED15 knockdown. **F**, **G** Transwell assays showed decreased migration and invasion of RCC cells with MED15 knockdown. **H** Western blot assay and qRT–PCR revealed that MED15 protein and mRNA were efficiently increased in RCC cells with MED15 overexpression. **I** Growth curves for 786-O cells (n = 3) and OS-RC-2 cells (n = 6) with MED15 overexpression detected by CCK-8 assay. **J** The colony formation assay showed an increased proliferation rate in RCC cells with MED15 overexpression on day ten after cultivation. **K**, **L** The wound healing assay indicated increased migration of RCC cells with MED15 overexpression. **M**, **N** Transwell assays showed increased migration and invasion of RCC cells with MED15 overexpression. The results are expressed as the mean ± standard deviation (SD). ** indicates *P* < 0.01; *** indicate*s P* < 0.001; **** indicates *P* < 0.0001. *RCC* renal cell carcinoma, *qRT–PCR* quantitative reverse transcription-polymerase chain reaction.

To determine the effect of MED15 on biological functions in ccRCC, we successfully knocked down MED15 expression in the 786-O and OS-RC-2 cell lines by transfecting cells with short hairpin RNA (shRNA) (Fig. 2A). A CCK-8 assay was performed and showed that the proliferation rate of 786-O and OS-RC-2 cells was significantly repressed with MED15 knockdown (Fig. 2B). The colony formation assay indicated the same results (Fig. 2C). The wound healing assay indicated that MED15 knockdown suppressed the flattening and spreading of 786-O (Fig. 2D) and OS-RC-2 cells (Fig. 2E). Transwell assays were performed to assess migration and invasion. The results showed that migratory ability and invasiveness were suppressed in MED15 knockdown compared with control 786-O (Fig. 2F) and OS-RC-2 cells (Fig. 2G). Moreover, we successfully constructed 786-O and OS-RC-2 cell lines with MED15 overexpression via transfection of overexpression lentivirus (Fig. 2H). The CCK-8 assay and colony formation assay indicated that the proliferation of MED15-overexpressing cells were increased (Fig. 2I, J). Transwell and wound healing assays showed markedly enhanced migratory ability and invasiveness of MED15-overexpressing 786-O (Fig. 2K, M) and OS-RC-2 cells (Fig. 2L, N). Overall, MED15 promoted the progression of ccRCC.

### MED15 functioned as a key molecule promoting lipid accumulation in ccRCC

**Fig. 3:**
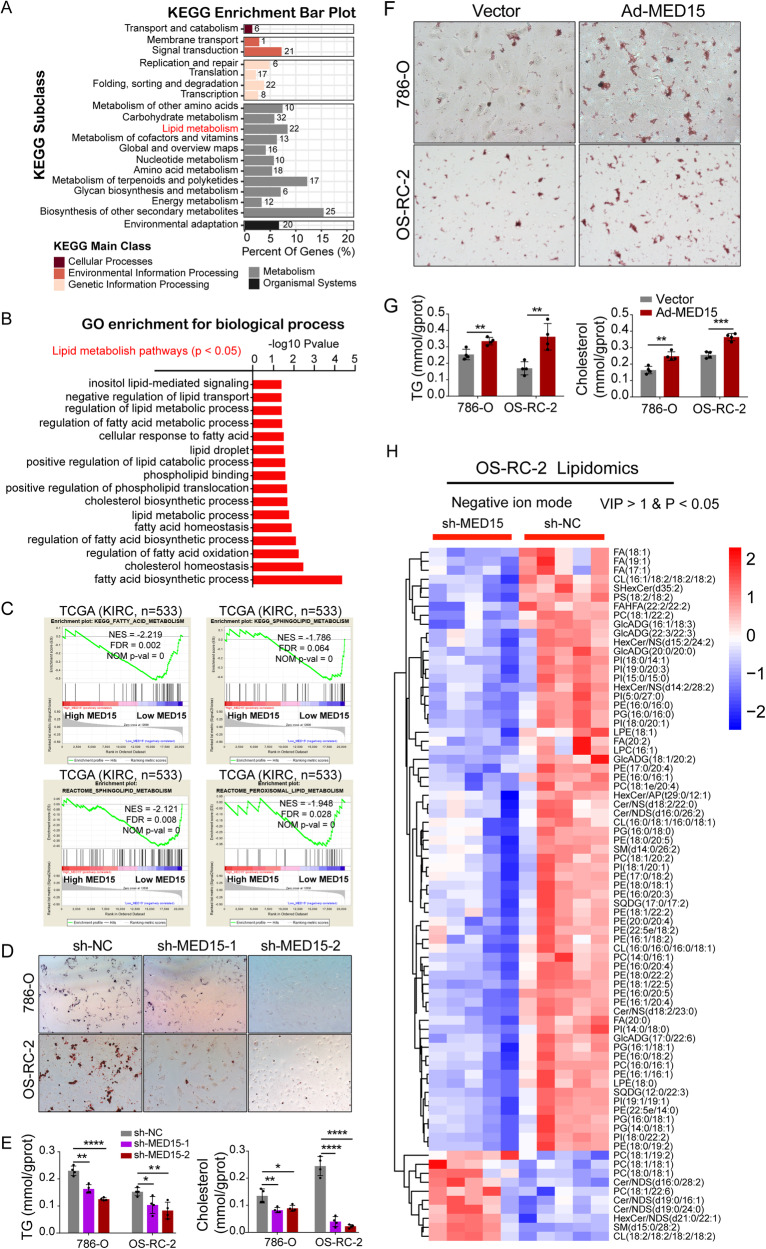
MED15 promoted lipid accumulation in ccRCC. **A** The results of the KEGG enrichment analysis of differentially expressed genes identified based on analysis of transcriptome sequencing data of OS-RC-2 cells with MED15 knockdown. **B** Enriched biological processes related to lipid metabolism with *P* < 0.05 according to the GO enrichment analysis. **C** GSEA of TCGA data showed that lipid metabolism terms were associated with the mRNA expression of MED15 according. FDR < 25% and *p* < 0.05 were used as criteria for determining statistical significance. **D** Oil Red O staining in RCC cells with MED15 knockdown. **E** The TG and cholesterol contents were measured in RCC cells with MED15 knockdown (n = 4). **F** Oil Red O staining in RCC cells overexpressing MED15. **G** The TG and cholesterol contents were measured in RCC cells overexpressing MED15 (n = 4). **H** The differentially expressed lipid components in negative ion mode were detected by LC/MS lipidomics assay in OS-RC-2 cells with or without MED15 knockdown (n = 5). The results are expressed as the mean ± standard deviation (SD). * indicates *P* < 0.05; ** indicates *P* < 0.01; *** indicate*s P* < 0.001; **** indicates *P* < 0.0001. KEGG Kyoto Encyclopedia of Genes and Genomes, GO Gene Ontology, GSEA gene set enrichment analysis, TCGA The Cancer Genome Atlas, FDR false discovery rate, TG triglyceride, RCC renal cell carcinoma; LC/MS liquid chromatography/mass spectrometry.

Abnormal lipid accumulation has been proven to be a key factor promoting the progression of ccRCC [12]. A study showed that MED15 is required for fatty acid homeostasis in *Caenorhabditis elegans* [19]. Whether MED15 is involved in lipid homeostasis in human diseases, including cancers, remains unknown. To explore the effect of MED15 on lipid accumulation in ccRCC, OS-RC-2 cells with MED15 knockdown were subjected to transcriptome sequencing. The results of KEGG enrichment analysis showed that categories related to lipid metabolism were significantly enriched (Fig. 3A). The enriched biological process terms from the GO enrichment analysis showed that MED15 was related to not only the malignant behavior of tumor cells (Fig. S2A) but also many lipid metabolism processes (Fig. 3B). GSEA also showed that MED15 was highly associated with lipid metabolism in the ccRCC TCGA dataset (Fig. 3C, Fig. S2B). To confirm this result, an oil red O staining assay was performed, and TGs and cholesterol were measured as visual indicators of intracellular lipids in ccRCC. The results of the oil red O staining assay showed a significant reduction in lipid deposition in stable MED15 knockdown cells (Fig. 3D). TGs and cholesterol were reduced in stable MED15 knockdown cells (Fig. 3E). However, cells with stable overexpression of MED15 exhibited relatively higher lipid deposition, as suggested by the oil red O staining assay (Fig. 3F), and higher TGs and cholesterol (Fig. 3G). Moreover, we used a LC/MS lipidomics assay to compare the relative changes in lipid metabolites in OS-RC-2 cells with or without MED15 knockdown. Principal component analysis (PCA) based on all detected lipid expression profiles showed different distribution patterns for cells with or without MED15 knockdown in negative ion mode (Fig. S2C) and positive ion mode (Fig. S2D). We found that the majority of the components of lipid droplets were significantly downregulated in stable MED15 knockdown cells in negative ion mode (Fig. 3H) and positive ion mode (Fig. S2E). These results suggested that MED15 promotes lipid accumulation in ccRCC.

### HIF-2α directly bound the MED15 promoter and stimulated its activity in ccRCC

**Fig. 4:**
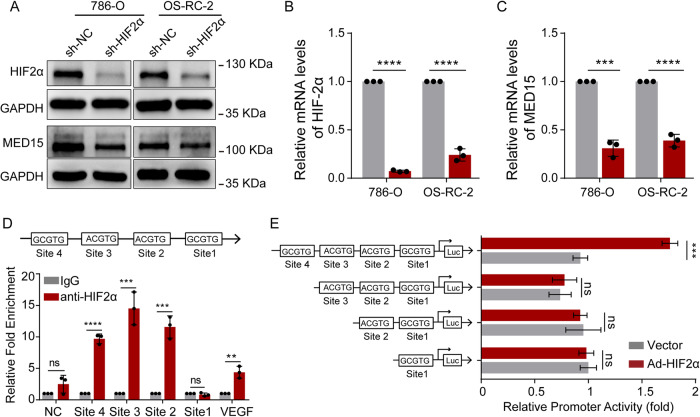
HIF-2α directly regulates MED15 transcription in ccRCC. **A**–**C** The protein and mRNA levels of HIF-2α and MED15 in RCC cells with HIF-2α knockdown. **D** Chromatin immunoprecipitation assays were performed on OS-RC-2 cells using anti-HIF-2α antibody followed by SYBR Green quantitative real-time polymerase chain reaction (qRT−PCR) for assessment of vascular endothelial growth factor (VEGF) and MED15 hypoxia response element (HRE) sites. **E** Luciferase reporter assays were performed in 293T cells cotransfected with truncated plasmids and HIF-2α expression plasmids or empty plasmid. After 24 h, Firefly luciferase and Renilla luciferase activities were measured using a dual-luciferase reporter assay, and the ratio of the Firefly/Renilla values was calculated (n = 3). The results are expressed as the mean ± standard deviation (SD). “ns” indicates *P* > 0.05; ** indicates *P* < 0.01; *** indicate*s P* < 0.001; **** indicates *P* < 0.0001.

HIF-2α plays a key role in lipid accumulation in ccRCC [12, 13]. To confirm the above results, an oil red O staining assay was performed and showed a significant reduction in lipid deposition in stable HIF-2α knockdown cells (Fig. S2F), revealing important roles of HIF-2α in lipid accumulation in ccRCC. In addition, sequencing of stable HIF-2α knockdown renal cancer cells identified MED15, which was proven to promote lipid accumulation in ccRCC. However, the mechanism by which HIF-2α regulates MED15 expression and whether MED15 is involved in HIF-2α-mediated lipid deposition remained to be determined. To address the first aspect, stable HIF-2α knockdown cells were constructed, and decreased HIF-2α expression at the protein level (Fig. 4A) and mRNA level was verified (Fig. 4B). The knockdown of HIF-2α significantly decreased MED15 protein (Fig. 4A) and mRNA levels (Fig. 4C) in both 786-O and OS-RC-2 cells. To gain insight into the mechanism of the regulation of MED15 expression by HIF-2α, the 1200 bp promoter sequence of the MED15 transcriptional start site was analyzed for potential hypoxia response elements (HREs) containing the consensus sequence (A/G)CGTG [23]. ChIP assays showed that HIF-2α bound to MED15 at site 2, site 3 and site 4, which were comparable to the binding sites indicating established HREs in the VEGF gene (Fig. 4D). Luciferase reporter assays were performed in 293 T cells cotransfected with truncated plasmids and HIF-2α expression plasmids or empty plasmid. We found that MED15 promoter (including site4, site 3, site 2, and site 1) markedly increased the promoter activity of MED15 induced by HIF-2α overexpression. The increased luciferase activity mediated by HIF-2α overexpression was significantly reversed after MED15 promoter site 4 was excised, while the removal of site 3, or 2, or 1 had no significant reverse effect (Fig. 4E). These results suggested that HIF-2α increases MED15 expression by binding to site 4 in the promoter sequence of MED15.

### MED15 promoted lipid accumulation by promoting the activation of SREBP-dependent lipid biosynthesis enzymes

**Fig. 5:**
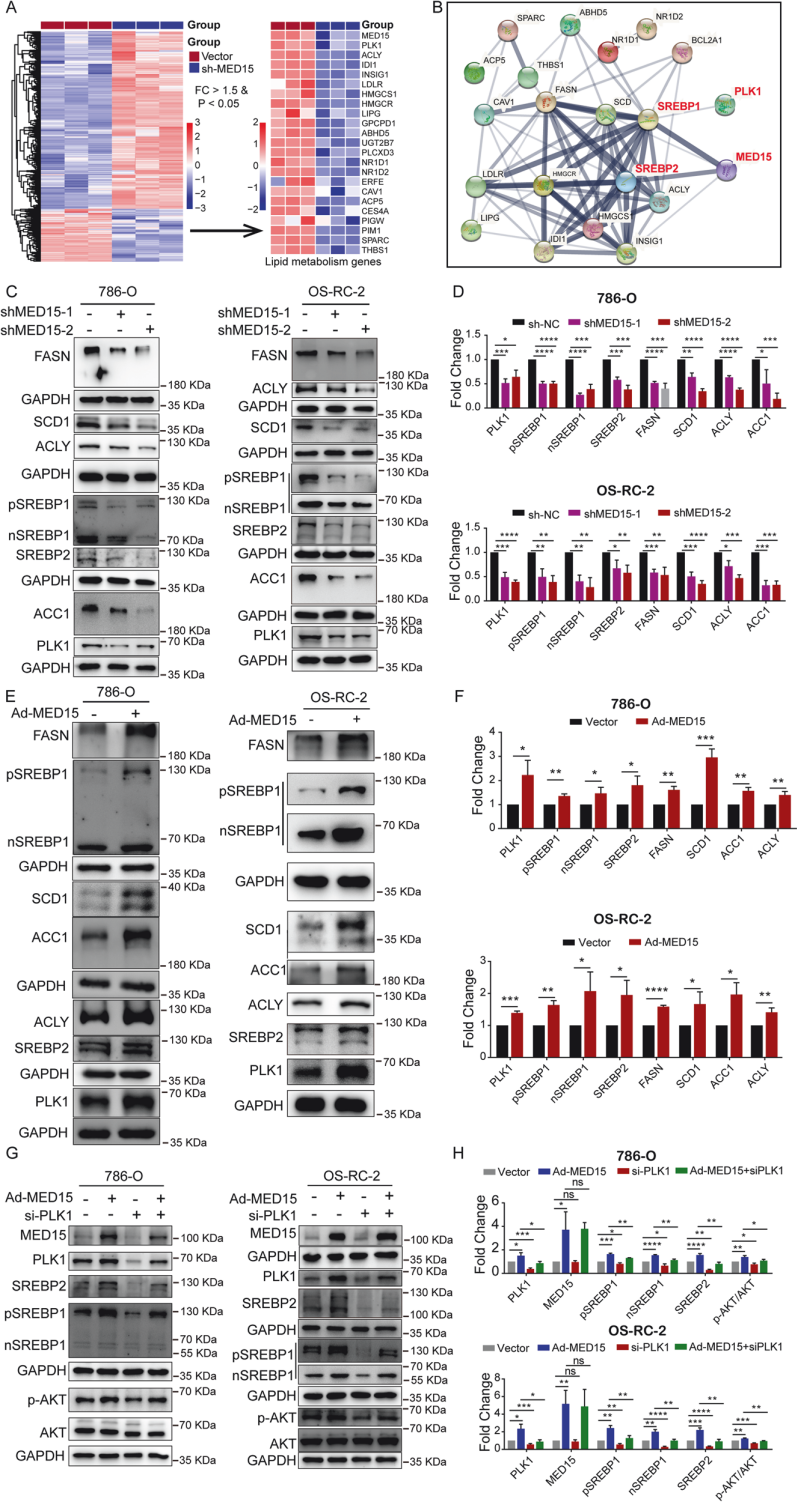
MED15 promoted activation of the SREBP pathway. **A** Downregulated lipid metabolism genes identified in transcriptome sequencing data of OS-RC-2 cells with MED15 knockdown. **B** Functional protein association networks for these downregulated lipid metabolism genes were constructed in the STRING database. **C**, **D** Western blot assays showed decreased protein levels of SREBP1, SREBP2, PLK1 and SREBP-dependent lipid biosynthesis enzymes (FASN, ACC1, ACLY, and SCD1) in RCC cells with MED15 knockdown. **E**, **F** Western blot assays showed increased protein levels of SREBP1, SREBP2, PLK1 and SREBP-dependent lipid biosynthesis enzymes (FASN, ACC1, ACLY, and SCD1) in RCC cells overexpressing MED15. **G**, **H** PLK1 knockdown eliminated the effects of MED15 overexpression on SREBP1 and SREBP2 expression. The results are expressed as the mean ± standard deviation (SD). “ns” indicates *P* > 0.05; * indicates *P* < 0.05; ** indicates *P* < 0.01; *** indicate*s P* < 0.001; **** indicates *P* < 0.0001.

To gain insight into the mechanism by which MED15 promotes lipid accumulation in ccRCC, the lipid metabolism genes downregulated in stable MED15 knockdown cells were determined based on the RNA sequencing results (Fig. 5A). Then, we used the online STRING database (http://string-db.org/) to visualize the functional protein association networks of these downregulated lipid metabolism genes [24]. We found that MED15 was highly associated with the SREBP-mediated lipid metabolism pathway (Fig. 5B). Previous studies have shown that MED15 is a coactivator and can interact directly with SREBPs, which are critical for lipid biosynthesis in *C. elegan*s [18]. Whether MED15 promotes lipid deposition in ccRCC through activation of SREBPs has not been clarified. To test this hypothesis, the correlations of MED15 with SREBP1 and SREBP2 were evaluated with TCGA-KIRC expression data. We found that MED15 had a significant positive correlation with the expression of SREBP1 (Fig. S3A), while MED15 was not correlated with the expression of SREBP2 (Fig. S3B). Then, quantitative real-time PCR (qRT–PCR) and western blotting assays were used to clarify the association between MED15 and key SREBP-dependent lipid biosynthesis enzymes in 786-O and OS-RC-2 cells with MED15 overexpression and knockdown. The qRT–PCR results showed significantly lower mRNA levels of key SREBP-dependent lipid biosynthesis enzymes (SREBP1, SREBP2, FASN, SCD1, ACLY, and ACC1) in stable MED15 knockdown 786-O (Fig. S3C) and OS-RC-2 cells (Fig. S3D). The results of the western blotting assays in stable MED15 knockdown 786-O and OS-RC-2 cells were in line with the qRT–PCR results (Fig. 5C, D). We found higher expression levels of key SREBP-dependent lipid biosynthesis enzymes (SREBP1, SREBP2, FASN, SCD1, ACLY, and ACC1) at both the mRNA level (Fig. S3E, F) and protein level (Fig. 5E, F) in MED15-overexpressing cells. These results suggested that MED15 promotes the activation of SREBPs.

### MED15 upregulated the expression of PLK1 and further promoted the transcriptional activation of SREBPs

The N-terminus of MED15 has a structure similar to the KIX domain of CREB-binding protein (CBP), which interacts only with SREBPs to promote the transcription of SREBP-responsive genes [18]. We found that MED15 knockdown resulted in a decrease in SREBP-targeted gene (FASN, SCD1, ACLY, and ACC1) expression, and MED15 overexpression increased the expression of SREBP-targeted genes (FASN, SCD1, ACLY, and ACC1) in ccRCC. Moreover, MED15 knockdown also decreased the expression of SREBP1 and SREBP2; MED15 overexpression increased the expression of SREBP1 and SREBP2. These results suggested that MED15 not only interacts with SREBPs but also may promote the activation of SREBPs through other pathways. From the RNA sequencing results, we found a significant decrease in PLK1 expression in stable MED15 knockdown cells (Fig. 5A). STRING analysis showed that PLK1 was also associated with the SREBP-mediated pathway (Fig. 5B). Therefore, we detected the expression of PLK1 in cells with MED15 overexpression and knockdown. We found decreased expression of PLK1 in stable MED15 knockdown 786-O and OS-RC-2 cells at both the mRNA (Figs. S3C, 3D) and protein levels (Fig. 5C, D). Higher expression of PLK1 was found in MED15-overexpressing 786-O and OS-RC-2 cells at both the mRNA (Figs. S3E, 3F) and protein levels (Fig. 5E, F). These results suggested that MED15 promoted the transcriptional activation of PLK1. Zhang et al. reported that PLK1 could activate the PI3K/AKT/mTOR pathway to elevate SREBP-dependent expression of key lipid biosynthesis enzymes in castration-resistant prostate cancer [25]. These results led us to speculate that PLK1 might be involved in MED15-mediated transcriptional activation of SREBPs. To test this hypothesis, we detected changes in the protein levels of SREBP1 and SREBP2 in PLK1 knockdown cells by transfecting PLK1 small interfering RNA (siRNA) into cells overexpressing MED15 (Fig. 5G, H). The results showed that the expression levels of SREBP1 and SREBP2 were decreased in PLK1 knockdown cells compared with control cells, suggesting that PLK1 can independently promote the expression of SREBPs in ccRCC. In addition, the expression of SREBP1 and SREBP2 was decreased in MED15-overexpressing cells with PLK1 knockdown compared with MED15-overexpressing cells, suggesting that PLK1 was responsible for coordinating and driving MED15-mediated transcriptional activation of SREBPs. These results suggested that MED15 upregulates the expression of PLK1, further promoting the transcriptional activation of SREBPs.

### MED15 was required for the lipid accumulation and pro-carcinogenic effects induced by HIF-2α

**Fig. 6:**
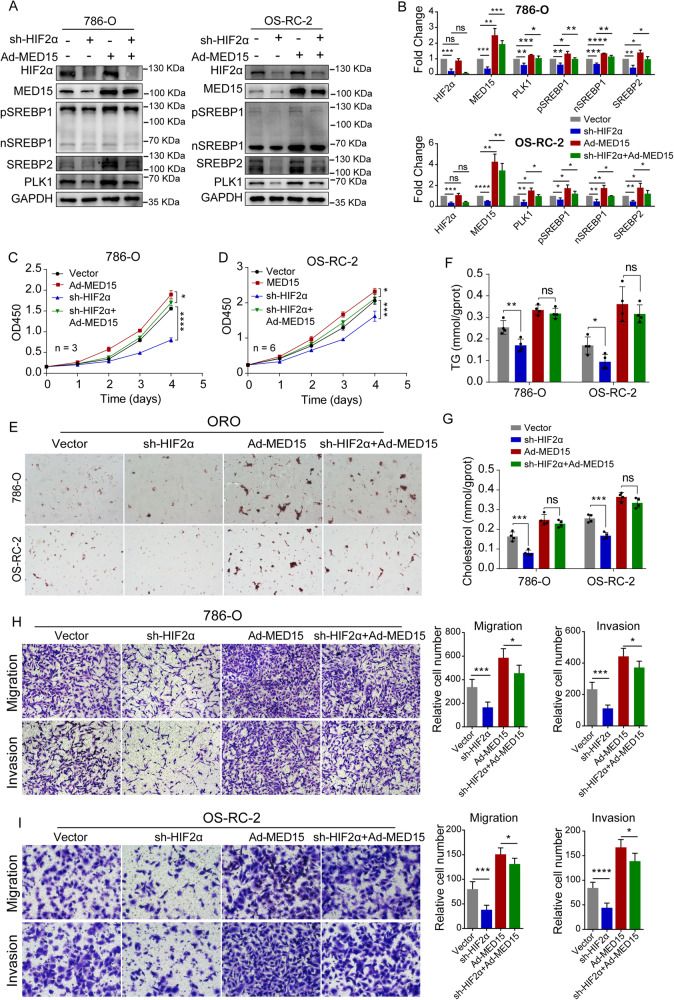
Overexpression of MED15 rescued the effects of HIF-2α knockdown on the lipid accumulation, migration, and invasion of RCC cells and tumor growth. **A**, **B** RCC cells with stable HIF-2α knockdown and MED15 overexpression. Western blot assays were performed to detect the expression of HIF-2α, MED15, PLK1, SREBP1 and SREBP2. **C**, **D** Growth curves for 786-O cells (n = 3) and OS-RC-2 cells (n = 6) with stable HIF-2α knockdown and MED15 overexpression detected by CCK-8 assay. **E** Oil Red O staining in RCC cells with stable HIF-2α knockdown and MED15 overexpression. **F**, **G** The TG and cholesterol contents were measured in RCC cells with stable HIF-2α knockdown and MED15 overexpression (n = 4). **H**, **I** Transwell assays of migration and invasion were performed with the transfected cell lines. The results are expressed as the mean ± standard deviation (SD). “ns” indicates *P* > 0.05; * indicates *P* < 0.05; ** indicates *P* < 0.01; *** indicate*s P* < 0.001; **** indicates *P* < 0.0001.

Given that MED15 was identified as a direct target of transcriptional activation by HIF-2α through promoter region activation, we reasoned that MED15 was required for the lipid accumulation and the procarcinogenic effects induced by HIF-2α in ccRCC. To test this hypothesis, we detected the changes in key proteins of the SREBP pathway (SREBP1, SREBP2 and PLK1) affected by MED15-mediated transcriptional activation via transfection of MED15 overexpression lentivirus in cells with HIF-2α knockdown. To complete this experiment, we constructed four groups of cell lines for rescue experiments, including sh-HIF-2α control and MED15 expression lentivirus control vector cells, sh-HIF-2α and MED15 expression lentivirus control vector cells, sh-HIF-2α control and MED15 expression lentivirus cells, and sh-HIF-2α and MED15 expression lentivirus cells (Fig. 6A, B). Functional rescue experiments, including CCK-8 assays, transwell assays, oil red O staining, and western blotting assays, were performed. The results showed that the MED15-mediated transcriptional activation of SREBP pathway members (SREBP1, SREBP2 and PLK1) was decreased in HIF-2α knockdown cells compared with control cells, suggesting that HIF-2α can independently promote the expression of SREBP1, SREBP2 and PLK1 in ccRCC. These results somewhat agree with previously published studies [26, 27]. In addition, the expression levels of SREBP1, SREBP2 and PLK1 were increased in HIF-2α knockdown cells with MED15 overexpression compared with HIF-2α knockdown cells, suggesting that MED15-mediated transcriptional activation of the SREBP pathway is regulated by HIF-2α (Fig. 6A, B). The CCK-8 assay showed that the proliferation of cells with HIF-2α knockdown was significantly inhibited compared with that of the vector group. Moreover, MED15 overexpression significantly alleviated the inhibition of cell proliferation induced by HIF-2α knockdown (Fig. 6C, D). Oil red O staining showed that MED15 overexpression significantly alleviated the inhibition of lipid deposition induced by HIF-2α knockdown (Fig. 6E). The detection of TG and cholesterol showed the same changes in lipid deposition in ccRCC cells during this process. The inhibition of lipid deposition induced by HIF-2α knockdown was significantly alleviated in MED15-overexpressing cells (Fig. 6F, G). Transwell assays showed that MED15 overexpression significantly alleviated the inhibition of migratory ability and invasiveness induced by HIF-2α knockdown (Fig. 6H, I). These results suggested that MED15 is regulated by HIF-2α and is required for the lipid accumulation and procarcinogenic effects induced by HIF-2α in ccRCC.

### MED15 promoted ccRCC progression and lipid accumulation in vivo

**Fig. 7:**
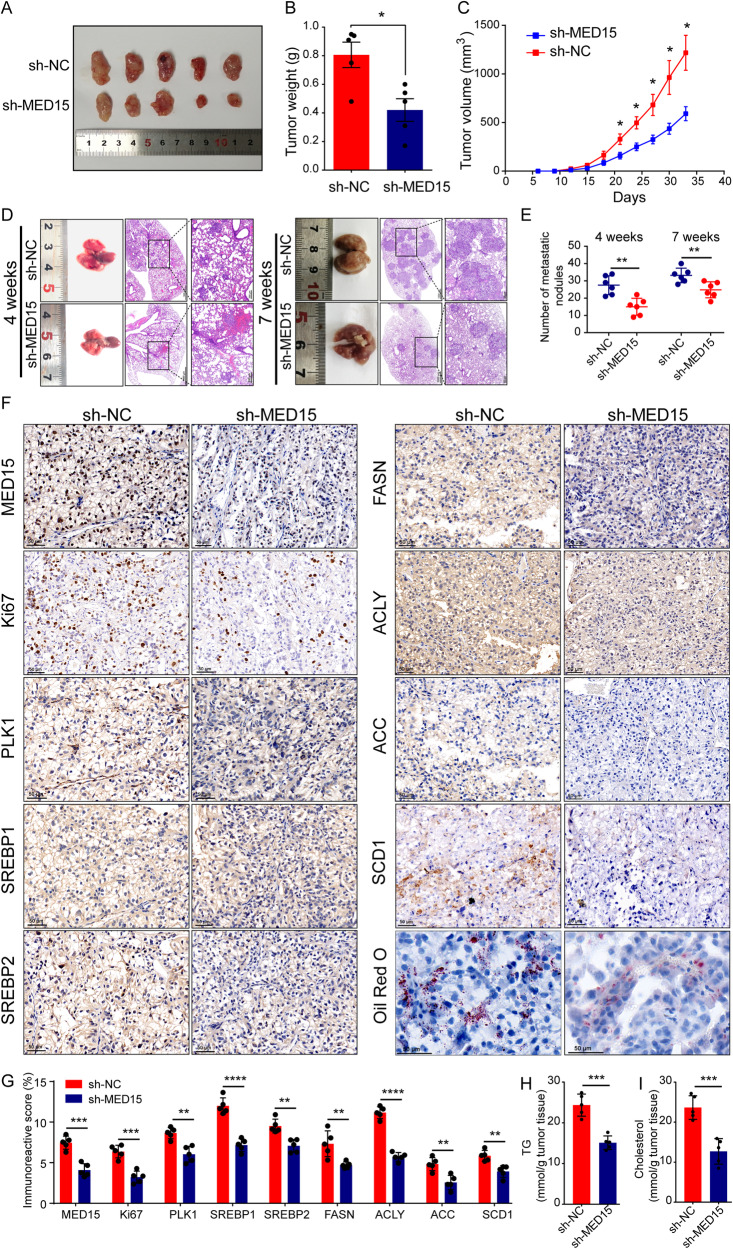
MED15 knockdown inhibited tumor progression and lipid accumulation in ccRCC in vivo. **A**–**C** OS-RC-2 cells with stable MED15 knockdown were implanted subcutaneously into the flanks of nude mice, and tumor size was measured every three days. Both tumor weight and tumor volumes were reduced in cells with stable MED15 knockdown (n = 5). **D** Macroscopic view and H&E staining of lung from the tail vein metastasis model at 4 weeks and 7 weeks. **E** The numbers of metastatic nodules in the lung were reduced in cells with stable MED15 knockdown at 4 weeks and 7 weeks (n = 6). **F**, **G** Immunohistochemical staining was performed to detect the levels of MED15, Ki67, PLK1, and SREBP-dependent lipid biosynthesis enzymes (SREBP1, SREBP2, FASN, ACC1, ACLY, and SCD1) in the subcutaneous tumorigenesis nude mouse model (n = 5). **H**, **I** The TG and cholesterol contents were measured in the subcutaneous tumorigenesis nude mouse model (n = 5). The results are expressed as the mean ± standard deviation (SD). “ns” indicates *P* > 0.05; * indicates *P* < 0.05; ** indicates *P* < 0.01; *** indicate*s P* < 0.001; **** indicates *P* < 0.0001.

To investigate the role of MED15 in tumor growth in vivo, OS-RC-2 cells with stable MED15 knockdown were implanted subcutaneously into the flanks of nude mice. Tumor size was measured every three days, starting at day 6 and continuing until day 33. The results showed that both tumor weight (Fig. 7A, B) and tumor volumes (Fig. 7C) were significantly reduced in the MED15 knockdown group. The tail vein metastasis model was used to assess the effect of MED15 on tumor metastasis in vivo. The mice were sacrificed, and their lungs were dissected at 4 weeks and 7 weeks after injection. The metastatic nodules on the lung surfaces of the mice were significantly reduced after MED15 knockdown at both 4 weeks and 7 weeks (Fig. 7D). H&E staining was used to confirm the numbers of metastatic nodules (Fig. 7D). The results showed that MED15 knockdown significantly reduced the lung metastasis of tumor cells (Fig. 7E). IHC staining showed that MED15, PLK1 and SREBP-dependent expression of key lipid biosynthesis enzymes (SREBP1, SREBP2, FASN, ACLY, ACC1, SCD1) was significantly reduced in the sh-MED15 group, which was consistent with the results in cell lines (Fig. 7F, G). Ki67 staining was significantly decreased in the sh-MED15 group, suggesting that MED15 knockdown inhibits cell proliferation (Fig. 7F, G). Oil red O staining and TG and cholesterol staining were used as indicators of lipid content in the subcutaneous xenograft tumor model. We found decreased lipid accumulation in mice in the sh-MED15 group (Fig. 7F, H, I). These results suggested that MED15 knockdown inhibits lipid accumulation and tumor progression in a subcutaneous xenograft tumor model, which was consistent with the results in cells.

### MED15 acts an oncogene in ccRCC and predicts poor prognosis for ccRCC patients

**Fig. 8:**
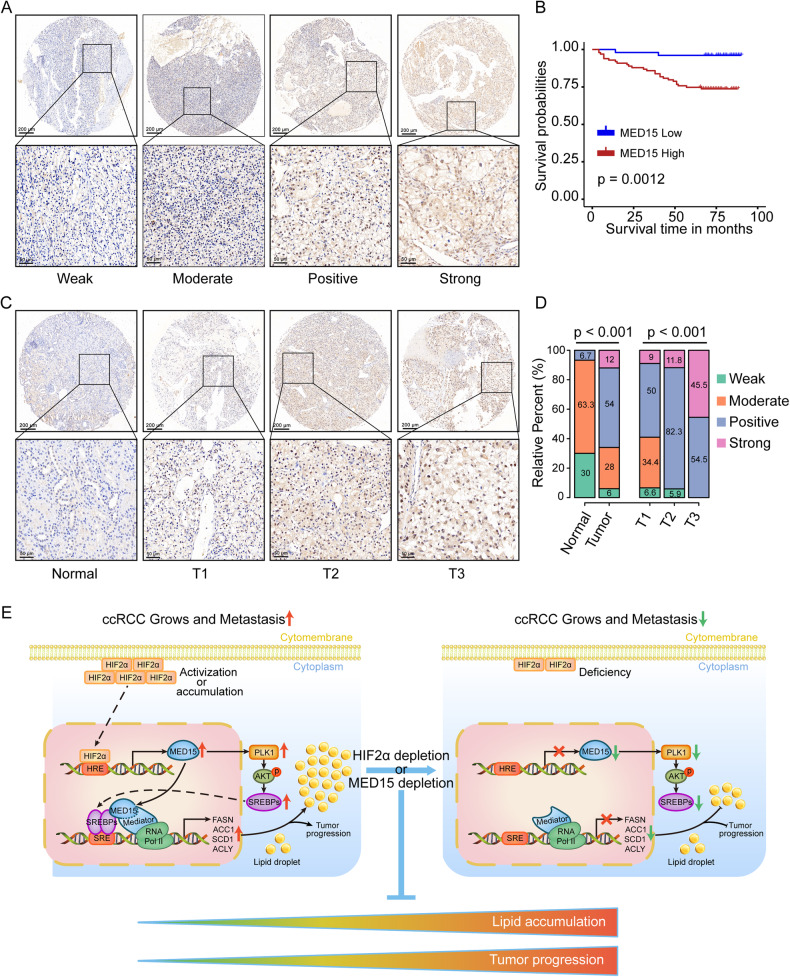
MED15 expression was positively associated with tumor stage and predicted poor prognosis in ccRCC specimens. **A** Examples of MED15 staining intensity (weak, moderate, positive, and strong). **B** Kaplan–Meier survival curves showing poor prognosis for ccRCC patients in the high MED15 expression group. **C**, **D** MED15 expression was increased in ccRCC specimens and was positively associated with tumor stage. **E** Schematic diagram depicting a proposed model for the major mechanism underlying the effects of HIF-2α dependent MED15 expression on the regulation of ccRCC progression and lipid accumulation.

To further determine the clinical significance and prognostic value of MED15 in ccRCC, the expression of MED15 in 150 ccRCC and 30 normal tissue specimens was analyzed (Table S4). An example of MED15 staining intensity (weak, moderate, positive, and strong) is shown in Fig. 8A. We found that the expression of MED15 in ccRCC can be divided into two categories: low MED15 (weak and moderate) and high MED15 (positive and strong). Kaplan–Meier survival curves showed that patients in the high MED15 group had a worse overall survival rate than those in the low MED15 group (Fig. 8B), suggesting that MED15 may be a potentially valuable prognostic biomarker for ccRCC. In addition, we found higher expression of MED15 in tumors, and this higher expression was associated with higher tumor stage (Fig. 8C, D). These results suggested that MED15 acts as an oncogene in ccRCC and has potential as a biomarker of prognosis in ccRCC.

## Discussion

Our work demonstrates that MED15 promotes lipid deposition and tumor progression in renal carcinoma by altering lipid synthesis pathways. MED15, a transcription cofactor, interacts directly with SREBPs and RNA polymerase II to regulate SREBP-dependent expression of key lipid biosynthesis enzymes. MED15 can also activate the PLK1/AKT pathway to further promote the transcriptional activation of SREBPs. We identified MED15 as a direct target of transcriptional activation induced by HIF-2α subunits, which occurs through promoter region activation. Depletion of HIF-2α or MED15 in ccRCC inhibited the formation of lipid droplets and tumor progression (Fig. 8E). Accordingly, overexpression of MED15 in HIF-2α knockdown cells restored lipid deposition and malignant tumor behavior phenotypes, suggesting that MED15-mediated lipid deposition and tumor progression in ccRCC are regulated by HIF-2α. We also found higher expression of MED15 in ccRCC than in normal kidney tissues in clinical samples, and the expression level of MED15 was positively correlated with tumor stage, suggesting that MED15 has the potential to be a biomarker of prognosis in ccRCC.

Metabolic reprogramming is a hallmark event of tumor cells that can drive tumor initiation and progression [28, 29]. Lipid accumulation, an important phenomenon in metabolic reprogramming, is usually upregulated in tumor cells and promotes a selective advantage to tumor cells [13]. Lipid accumulation not only affects the growth and survival of cancer cells but also regulates the processes of cell diffusion and metastasis [30, 31]. Specifically, an imbalance in lipid metabolism is a typical feature of ccRCC, which shows large amounts of glycogen and lipid accumulation [11]. Dysregulation of lipid metabolism is associated with ccRCC carcinogenesis, aggressiveness, cancer stem cell invasiveness, compromised efficacy of sunitinib, and poor prognosis [12, 13, 32, 33]. Therefore, identification of the mechanisms of lipid metabolism reprogramming in ccRCC can provide novel targets for the prevention and treatment of ccRCC. Horiguchi et al. identified that FASN expression was increased in ccRCC and that increased FASN expression was an indicator of tumor aggressiveness and poor prognosis for ccRCC patients [33]. Pharmacological inhibition of FASN inhibited tumor growth and was an effective strategy for treating ccRCC [33]. Roemeling et al. identified that SCD1 expression was increased in ccRCC, and pharmacologic inhibition of SCD1 inhibited tumor cell proliferation and induced apoptosis [34]. Qiu et al. identified that the lipid droplet coat protein PLIN2 can promote lipid storage, endoplasmic reticulum homeostasis, and tumor progression in ccRCC [12]. PLIN2 depletion decreased lipid droplets, enhanced tumor cell cytotoxic endoplasmic reticulum stress, and inhibited the tumorigenic capacity of xenografts [12]. Du et al. identified that CPT1A, a rate-limiting component of mitochondrial fatty acid transport, can inhibit lipid deposition, cell survival and tumor growth in ccRCC [13]. Restoring the function of CPT1A in ccRCC might be a new therapeutic strategy for ccRCC [35]. However, studies on lipid metabolism in ccRCC are limited, and key targets that mediate lipid accumulation in ccRCC are poorly understood.

The mediator complex, as a coactivator, forms a bridge between gene-specific transcription factors and RNA polymerase II to play a role in many processes regulating gene transcription [36]. Gene transcriptional activation is the key to the regulation of gene expression and is associated with developmental diseases and cancer [37]. MED15, a subunit of the mediator complex, has been identified to be associated with multidrug resistance, obesity, and tumor aggressiveness [16, 38]. MED15 acts as an oncogene in numerous types of cancer, including hepatocellular carcinoma [39], prostate cancer [40], head and neck squamous cell carcinoma [41], bladder carcinoma [42], and renal carcinoma [22], to promote tumor progression. In the present study, we demonstrated for the first time that MED15 is essential for lipid deposition in the human disease ccRCC. MED15 depletion reduced lipid accumulation and inhibited tumor progression in ccRCC. Structurally, the N-terminus of MED15 has a domain similar to the KIX domain of CBP [18]. The KIX domain of MED15 is highly selective in binding to transcription factors and only interacts with SREBPs [18]. The interaction between MED15 and SREBPs in ccRCC was fully verified in this study by transcriptome sequencing and other experiments. Transcriptome sequencing and bioinformatics analysis showed that MED15 was highly correlated with the SREBP pathway. MED15 knockdown in ccRCC inhibited the expression of key SREBP-dependent lipid biosynthesis enzymes (FASN, ACC1, ACLY, and SCD1), which subsequently led to a decrease in lipid accumulation. The phenotype in ccRCC is similar to that in *C. elegans* [18, 43]. These results indicate that the interaction between MED15 and SREBPs is highly conserved across different species and diseases. Inhibition of the interaction between MED15 and SREBPs might be a novel therapeutic strategy for the treatment of various diseases with aberrant lipid metabolism, such as obesity and cancers. Importantly, a novel compound, BF175, has been discovered and can block the binding of MED15 KIX to SREBPs, resulting in the inhibition of SREBP-dependent expression of key lipid biosynthesis enzymes [44]. BF175 has been shown to improve diet-induced obesity in mice [44]. Therefore, we hypothesize that BF175 can also improve lipid homeostasis and inhibit tumor progression in ccRCC. However, further animal and clinical studies are needed to confirm the therapeutic effect and clinical application potential.

SREBPs are key transcription factors that regulate the expression of enzymes required for cholesterol, fatty acid, triglyceride and phospholipid synthesis. Three isoforms of SREBPs exist in mammals: SREBP1a, SREBP1c, and SREBP2. SREBP1 is involved in lipid synthesis and energy storage, and SREBP2 is involved in cholesterol biosynthesis [45]. An imbalance in lipid metabolism induced by the SREBP pathway is required for cell survival and tumor growth, and targeting SREBPs is a novel strategy for therapy in lipid-related disease [45, 46]. In ccRCC, aberrant lipid accumulation induced by the SREBP pathway has been confirmed, and SREBP-dependent lipid accumulation may be important for tumor cell growth [27, 47, 48]. Lee et al. found that SREBP1c overexpression promotes lipogenesis and tumor cell proliferation, and RNF20 inhibits lipogenesis and cell cycle progression in ccRCC by inhibiting the SREBP-1c pathway [49]. Syafruddin et al. found that KLF6 promotes lipid accumulation in ccRCC as a regulator of both the transcription of lipid metabolism genes and the activation of SREBP1 and SREBP2 through the PDGFB-mTOR axis [27]. Shen et al. identified that E2F1 promotes lipid storage and cell cycle progression through the modulation of SREBP1 in ccRCC [48]. Our study identified MED15 as a coactivator of SREBPs that promotes lipid accumulation and tumor progression in ccRCC and directly interacts with SREBPs through its KIX domain.

In cancer cells, the AKT-mediated activation of SREBPs is the primary mechanism in lipid metabolism and is involved in tumor cell survival and tumorigenesis [45, 46]. Recent studies have shown that PLK1 promotes the stabilization of nuclear SREBP1 during mitosis, providing a link between lipid metabolism and cell proliferation [50]. PLK1 can activate the PI3K/AKT/mTOR pathway to elevate SREBP-dependent expression of key lipid biosynthesis enzymes in castration-resistant prostate cancer [25]. Based on our analysis of RNA sequencing and bioinformatics data, we found potential associations between PLK1 and the SREBP pathway in ccRCC. These results suggested that PLK1 might regulate the SREBP pathway in ccRCC. MED15 knockdown not only reduced the expression of SREBP-dependent lipid biosynthesis enzymes (FASN, ACC1, ACLY, and SCD1) but also decreased the expression of SREBP1 and SREBP2, suggesting that MED15 and SREBPs not only interact directly but also have other regulatory modes. PLK1 can provide a perfect explanation for this result. MED15 activates the PLK1/AKT pathway to further promote the transcriptional activation of SREBPs. Our results suggested that PLK1 is required for the transcriptional activation of SREBP1 and SREBP2 in cells with MED15 overexpression. Overall, our results offer a molecular rationale supporting a combinatorial treatment approach including PLK1 inhibitors and drugs targeting the SREBP pathway or MED15 in ccRCC.

HIF-2α is the most critical oncogene in ccRCC and is involved in angiogenesis and multiple other processes, including the cell cycle, cell proliferation, tumor metastasis, resistance to oxidative stress and tumor glucose metabolism [10, 26, 51]. The associations between HIF-2α and angiogenesis form the basis of current targeted therapy strategies for ccRCC patients, for example, sunitinib, which targets vascular endothelial growth factor (VEGF) and platelet-derived growth factor (PDGF), both of which are important downstream targets of HIF-2α [52, 53]. Recent studies have shown that HIF-2α is a key regulator of lipid metabolism and that HIF-2α knockdown results in a decrease in lipid accumulation in ccRCC cell lines [12, 13, 54]. Two genes, PLIN2 and CPT1A, have been implicated in HIF-2α-mediated lipid accumulation in ccRCC [12, 13]. The HIF-2α/PLIN2 lipid storage axis suppresses cytotoxic endoplasmic reticulum stress to promote lipid storage and tumor cell proliferation [12]. The HIF-2α/CPT1A lipid storage axis can inhibit fatty acid transport into mitochondria to promote lipid droplet formation and tumor growth [13]. Our studies showed that MED15 is another dominant factor for HIF-2α-dependent lipid storage. HIF-2α promotes MED15 transcriptional activation by binding directly to the MED15 promoter region, further promoting lipid accumulation and tumor growth. Moreover, HIF-2α inhibitors have been shown to effectively inhibit ccRCC tumor growth, but resistance to HIF-2α inhibitors develops rapidly [7, 8]. Therefore, exploring other downstream targets of HIF-2α may provide new strategies for the treatment of drug-resistant cancers. HIF-2α selectively enriches polyunsaturated lipids by activating the expression of HILPDA, contributing to ferroptosis susceptibility [55]. Our studies showed that therapeutic strategies including MED15 depletion or blockade of the binding of MED15 to SREBPs might provide alternative treatment options for these drug-resistant cancers.

In summary, we describe a molecular regulatory network that links MED15 to the lipid metabolism induced by the SREBP pathway and the classic HIF-2α pathway in ccRCC. Our results showed that MED15 promotes lipid accumulation and tumor progression in ccRCC. MED15 acted as a coactivator of SREBPs, directly interacting with SREBPs to promote the expression of SREBP-dependent lipid biosynthesis enzymes and the activation of SREBP1 and SREBP2 through the PLK1/AKT axis. MED15 was identified as a downstream target of HIF-2α, participating in HIF-2α-mediated lipid accumulation in ccRCC. Enhanced MED15 expression was critical for ccRCC growth and was a biomarker for poor prognosis. Efforts to develop specific inhibitors of MED15 or of MED15 binding to SREBPs as novel therapeutic agents for ccRCC may be warranted.

## Materials and methods

### Human samples

A tissue microarray (TMA) containing 150 ccRCC and 30 normal tissue specimens was purchased from Shanghai Outdo Biotech (HKidE180Su02). We also collected samples of ccRCC and adjacent normal renal tissues from the Department of Urology, The First Affiliated Hospital of Anhui Medical University (Hefei, China), between 2019 and 2021. Samples were immediately frozen in liquid nitrogen and then stored at −80 °C. The histological diagnosis of ccRCC was confirmed by two pathologists. Informed consent forms were signed by all patients. The present study conformed to the standards of the Declaration of Helsinki and was approved by the Ethics Committee of Human Research of The First Affiliated Hospital of Anhui Medical University (No. PJ2019-14-22).

### Cell culture and reagents

Renal cell lines, including 786-O, A498, OSRC-2, ACHN, and Caki-1, the human renal proximal tubular epithelial cell line HK-2, and 293T cells were obtained from the cell culture center of the Chinese Academy of Medical Sciences (Shanghai, China). Caki-1 cells were cultured in McCoy’s 5 A medium; ACHN and A498 cells were cultured in MEM; 786-O and OSRC-2 cells were cultured in RPMI 1640 medium; and HK-2 and 293 T cells were cultured in DMEM. All media were supplemented with 10% fetal bovine serum, and cells were maintained at 37 °C in a humidified atmosphere with 5% CO_2_.

### Plasmids and siRNA

The MED15 knockout lentivirus, MED15 expression lentivirus, and HIF-2α plasmid were purchased from GeneChem. The knockout lentivirus for HIF-2α was purchased from Hanbio Tech. The siRNA for PLK1 was purchased from RiboBio. All steps were executed according to the manufacturers’ instructions. Human HIF-2α (NM_001430) cDNA was amplified with PCR and cloned into the GV358 lentiviral vector. Human MED15 (NM_015889) cDNA was amplified with PCR and cloned into the GV661 lentiviral vector. The oligonucleotide sequences specifically targeting HIF-2α and MED15 were as follows: sh-HIF-2α: 5′-GCCACAGCATGGACATGAAGT-3′, sh-Control: 5′-TTCTCCGAACGTGTCACGTAA; sh-MED15-1: 5′-ACCAAACAGCAGTACCTAT-3′, sh-MED15-2: 5′-ACAAGAACGAAGACAGAAA-3′, sh-Control: 5′-TTCTCCGAACGTGTCACGT-3′. The si-PLK1 sequence was as follows: 5′-GCTCTTCAATGACTCAACA-3′. The detailed information for the vectors is shown in Table S5. For transient transfection, Lipofectamine 2000 (Invitrogen; Thermo Fisher Scientific, Inc.) was used for the transfection of plasmids and siRNAs.

### RNA sequencing

RNA sequencing for stable MED15 knockdown OS-RC-2 cells was performed by LC-BIO Biotech (Hangzhou, China). The mRNA results for RNA sequencing was showed in Table S6. Differential expression analysis was performed using the DESeq R package, and |fold change| > 1.5 and *p* value < 0.05 were set as the thresholds. Functional enrichment analysis, including Gene Ontology (GO) functional and Kyoto Encyclopedia of Genes and Genomes (KEGG) pathway enrichment analyses, was performed for differentially expressed genes. A *p* value < 0.05 was considered statistically significant.

### Mouse tumor growth and metastasis

Male 6-week-old BALB/c nude mice were purchased from the Nanjing Biomedical Research Institute of Nanjing University (Nanjing, China), and were randomly assigned to each group. Xenograft tumor models were established by subcutaneous injection of OS-RC-2 cells with a total of 2 × 10^6^ cells in 100 μl of saline. Each group contains five mice. Tumor growth was measured every three days for 5 weeks, and tumor volumes were calculated by the formula V = 1/2 L × W^2^. After the mice were sacrificed, the tumor weight was measured, and IHC staining was conducted as previously described. Tumor cell metastasis ability was evaluated through tail vein injection of a total of 5 × 10^6^ OS-RC-2 cells in 100 μl of saline. The mice were sacrificed at 4 weeks and 7 weeks after injection. Each group contains six mice. The weights of the mice were measured, and metastatic lung lesions were counted after staining with H&E. All the animals used were housed in the specific pathogen-free research animal facility of Anhui Medical University. All animal protocols were approved by the Committee for Animal Care and Use of the Animal Center of Anhui Medical University.

### RNA isolation and qRT-PCR

Total RNA was extracted using TRIzol reagent (Invitrogen, Carlsbad, CA). Then, qRT-PCR was performed, and an ABI7500 platform (Thermo Fisher Scientific, Massachusetts, USA) was used for measurement according to the manufacturer’s instructions. Total RNA was reverse transcribed into cDNA using a PrimeScriptTM RT reagent kit (Takara, Kusatsu, Japan), and quantitative PCR was performed with SYBR green master mix (Takara, Kusatsu, Japan). Gene expression was normalized to that of GAPDH. Primers were chemically synthesized by Sangon Biotech (Shanghai, China). The primer sequences are shown in Table S7.

### Western blotting assay

Total protein was extracted using RIPA protein lysis buffer (Beyotime Biotech, Jiangsu, China) with PMSF and complete protease and phosphatase inhibitor cocktail. Protein concentrations were determined by a BSA kit (Beyotime Biotech, Jiangsu, China). Fifty micrograms of protein was separated by gel electrophoresis and transferred to methylcellulose membranes. The membranes were blocked in 5% nonfat dried skimmed milk for 2 hours at room temperature and incubated overnight at 4 °C with the following primary antibodies: anti-HIF-2α (1:1000; #59973; CST), anti-MED15 (1:1000; 11566-1-AP; Proteintech), anti-SREBP1 (1:200; sc-13551; Santa Cruz), anti-SREBP2 (1:500; ab30682; Abcam), anti-FASN (1:1000; ab128870; Abcam), anti-ACC1 (1:1000; ab45174; Abcam), anti-ACLY (1:1000; ab40793; Abcam), anti-SCD1 (1:1000; #2794; CST), anti-PLK1 (1:1000; #4513; CST), Phospho-AKT (Ser473) (1:1000; AF0016; Affinity), AKT (1:1000; AF6261; Affinity), and anti-GAPDH (1:1000, #2118; CST). The membranes were incubated with secondary antibodies for 2 hours after being washed with tris-buffered saline and Tween 20 and were visualized by an enhanced chemiluminescence kit (Biological Industries, Israel).

### IHC assay

IHC was conducted according to the protocol of the Two-step IHC Kit (ZSGB-BIO). Briefly, 4 µm thick slides of paraffin-embedded tissue specimens were processed for antigen retrieval with citric acid buffer (0.01 M, pH 6.0), and endogenous peroxidase activity was quenched with 3% hydrogen peroxide solution. Then, the slides were blocked with 10% bovine serum albumin and incubated overnight at 4 °C with the following primary antibodies: anti-MED15 (1:50; 11566-1-AP; Proteintech), anti-SREBP1 (1:100; AF6283; Affinity), anti-SREBP2 (1:100; ab30682; Abcam), anti-FASN (1:50; DF6106; Affinity), anti-ACC1 (1:100; ab45174; Abcam), anti-ACLY (1:100; ab40793; Abcam), anti-SCD1 (1:100; #2794; CST), anti-PLK1 (1:50; DF7004; Affinity), and anti-Ki67 (1:200, BS-2130R; Bioss). The slides were incubated with biotinylated goat anti-rabbit IgG (1:200) for 1 h at room temperature. DAB (ZLI-0918, ZSBio, China) was used to visualize the immune complexes, and the sections were counterstained with hematoxylin. Immunostaining intensity was measured using ImageJ software (National Institutes of Health, Bethesda, MD) as previously described [56].

### Cell proliferation and colony formation assays

Cells (1 × 10^3^ per well) were plated in 96-well plates and cultured for 0 h, 24 h, 48 h, 72 h, and 96 h. Then, the cell proliferation rate was detected using a CCK-8 kit (Dojindo Molecular Technology, Japan) according to the manufacturer’s instructions. For the colony formation assay, a total of 1000 cells were plated in 6-well plates. After a period of cultivation, colonies were fixed with methanol for 15 min and visualized with 0.05% crystal violet staining.

### Wound healing and transwell assays

Cells were seeded in 6-well plates and pretreated using serum-free medium when they reached 80% confluence. Cells were scratched with a sterile 200-μl pipette tip, and images of wounds were captured at 0 h and 24 h. Cell migration and invasion assays were performed using a 24-well Transwell chamber with 8 μm pores (Costar, Bodenheim, Germany). For invasion assays, the upper wells were coated in Matrigel (diluted in serum-free medium 1:8; BD Biosciences). After 24 h of incubation, cells attached to the lower surface of the membrane were fixed in 100% methanol for 15 min and stained with 0.05% crystal violet. Six random fields were chosen for imaging under a microscope (Olympus, Japan). The cells on the bottom surface of the chamber were counted.

### Oil red O staining assay

Oil red dye was prepared with saturated oil red O and ultrapure water at a ratio of 2:3. Cells were rinsed with PBS twice and fixed in 10% formaldehyde for 30 min. The cells were then rinsed with 60% isopropanol for 5 min, stained with oil red dye for 30 min, and washed with water three times to remove the excess oil red dye. Images were captured with a microscope when the plate dried.

### Triglyceride (TG) and cholesterol measurement assays

TGs and cholesterol in cells and tissues were detected by using a TG assay kit (cat. A110-1-1, Bioengineering) and total cholesterol assay kit (cat. A111-1-1, Bioengineering) according to the manufacturer’s instructions. Briefly, cells or tissues were lysed using 2% Triton X-100 and an ultrasonic apparatus. Then, 2.5 µl of the above lysed solution was added to 250 µl of working solution, incubated at 37 °C for 10 minutes, and measured at 510 nm using a spectrometer. The protein concentration of the samples was detected using a BSA kit. The formula for calculation is shown below:

TG or cholesterol content = (Sample OD – Blank OD)/(Calibration OD – Blank OD) × Standard concentration/Protein concentration.

### Liquid chromatography/mass spectrometry (LC/MS) lipidomics assay

The same number of OS-RC-2 cells with and without MED15 knockdown were collected and stored at -80 °C for LC/MS lipidomics assays. Cell samples were frozen with liquid nitrogen, homogenized with MTBE and sonicated in an ice-water bath for 10 min. Then, the samples were incubated at −40 °C for 1 h and centrifuged at 3000 rpm at 4 °C for 15 min. Three hundred microliters of supernatant was vacuum dried. The dried samples were reconstituted in 100 μL of 50% methanol in dichloromethane by sonication on ice for 10 min. A total of 75 μL recombinant supernatant was put into a new sample bottle for LC/MS lipidomics analysis. The detection and analysis of lipid metabolites were completed by high-performance liquid chromatography (Biotree, Shanghai, China) using a UHPLC system (1290, Agilent Technologies) in both positive and negative ion modes. A Kinetex C18 column (2.1 × 100 mm, 1.7 μm, Phenomen) was employed in both positive and negative ion modes. For data analysis, the SIMCA16.0.2 software package (Sartorius Stedim Data Analytics AB, Umea, Sweden) was employed for multivariate analysis. Data were scaled and logarithmically transformed to minimize the impact of both noise and high variance of the variables. Then, PCA was carried out to visualize the distribution patterns of the samples. Supervised orthogonal projections to latent structures-discriminate analysis (OPLS-DA) was applied to visualize group separation and find significantly changed metabolites. The value of variable importance in the projection (VIP) of the first principal component in OPLS-DA analysis was obtained, and *p* values were calculated with two-tailed Student’s *t* test on the normalized peak areas. Metabolites with VIP > 1 and *p* < 0.05 were considered significantly changed metabolites.

### Chromatin immunoprecipitation (ChIP) assay

ChIP assays were performed using 786-O cells cultured in 10 cm plates with a Simple ChIP Enzymatic Chromatin IP Kit (#9002; CST) according to the manufacturer’s protocol. Lysates were immunoprecipitated with anti-HIF-2α (1:100; #59973; CST) and normal rabbit IgG. The purified DNA specimens were analyzed by qRT-PCR to amplify fractions of the MED15 promoter. The primer sequences are provided in Table S8.

### Dual luciferase reporter assay

The HIF-2α expression plasmids, MED15 promoter reporter plasmids and pRL-TK (Renilla luciferase) plasmids were cotransfected into 293T cells using Lipofectamine 2000 (Invitrogen) in 24-well plates. After 24 h, the cells were quantified using the dual-luciferase reporter assay (Promega, Madison, WI, USA). The activities of Firefly (F) and Renilla (R) luciferase were measured. The relative luciferase activity was calculated as F/R. MED15-related luciferase plasmids were purchased from GeneChem, and the specific construction sequences are shown in Table S9.

### Bioinformatics analysis

The mRNA expression data and clinical data (stage, grade, T stage, N stage, M stage, overall survival, DFS and survival status) for The Cancer Genome Atlas (TCGA) dataset were obtained from UCSC Xena (https://xenabrowser.net/), and the expression data were subjected to log2(x + 1) transformation and RSEM normalization. Normalized gene data from the GSE53757, GSE76351, GSE36895, and GSE46699 datasets were obtained from the Gene Expression Omnibus (GEO) (https://www.ncbi.nlm.nih.gov/geo/) database. The detailed information for these public datasets is shown in Table S10. The lipid metabolism gene set REACTOME_METABOLISM_OF_LIPIDS was directly downloaded from the MSigDB database (http://software.broadinstitute.org/gsea/msigdb/index.jsp). The specific genes in this gene set are listed in Table S11. Differentially expressed genes between ccRCC samples and adjacent normal tissues were obtained using thresholds of |fold change| > 1.7 and *p* value < 0.05. Overall survival-related genes were selected using univariate Cox regression analysis with a *p* value < 0.05. Gene set enrichment analysis (GSEA) was carried out to identify enriched pathways with criteria false discovery rate (FDR) < 0.25 and nominal *p* < 0.05.

### Statistical analysis

Statistical analysis was performed with GraphPad Prism version 7.0 software (GraphPad Software, San Diego, CA). We performed unpaired, two-tailed Student’s *t*-tests to compare two groups and paired Student’s *t*-tests to compare paired samples between two groups. One-way ANOVA with the Bonferroni post hoc test was performed to compare multiple groups. Kaplan–Meier curves and the log-rank test were performed to evaluate prognostic significance. ROC curves and AUC values were used to evaluate diagnostic utility. The associations between MED15 expression and clinical factors in ccRCC patients were evaluated using Pearson’s χ^2^ test. Gene expression correlations were estimated using Pearson’s correlation coefficient. The results are expressed as the mean ± standard deviation (SD). *P* < 0.05 was considered statistically significant. In the figures, “ns” indicates *P* > 0.05; * indicates *P* < 0.05; ** indicates *P* < 0.01; *** indicate*s P* < 0.001; and **** indicates *P* < 0.0001.

### Supplementary information

Supplementary figures and tables

Supplementary Tabls S6

Original Western blot strips

## Data Availability

The datasets used and/or analyzed during the current study are available from the first author or corresponding author on reasonable request.
