# Supplementary material for: MED15 is upregulated by HIF-2α and promotes proliferation and metastasis in clear cell renal cell carcinoma via activation of SREBP-dependent fatty acid synthesis
Source: Cell Death Discov. 2024 Apr 22;10:188. doi: 10.1038/s41420-024-01944-1 (PMC11035615; doi:10.1038/s41420-024-01944-1)
Supplement: Supplementary file 1 — Supplementary figures and tables [file 41420_2024_1944_MOESM1_ESM.pdf]

Supplementary Figures and Tables

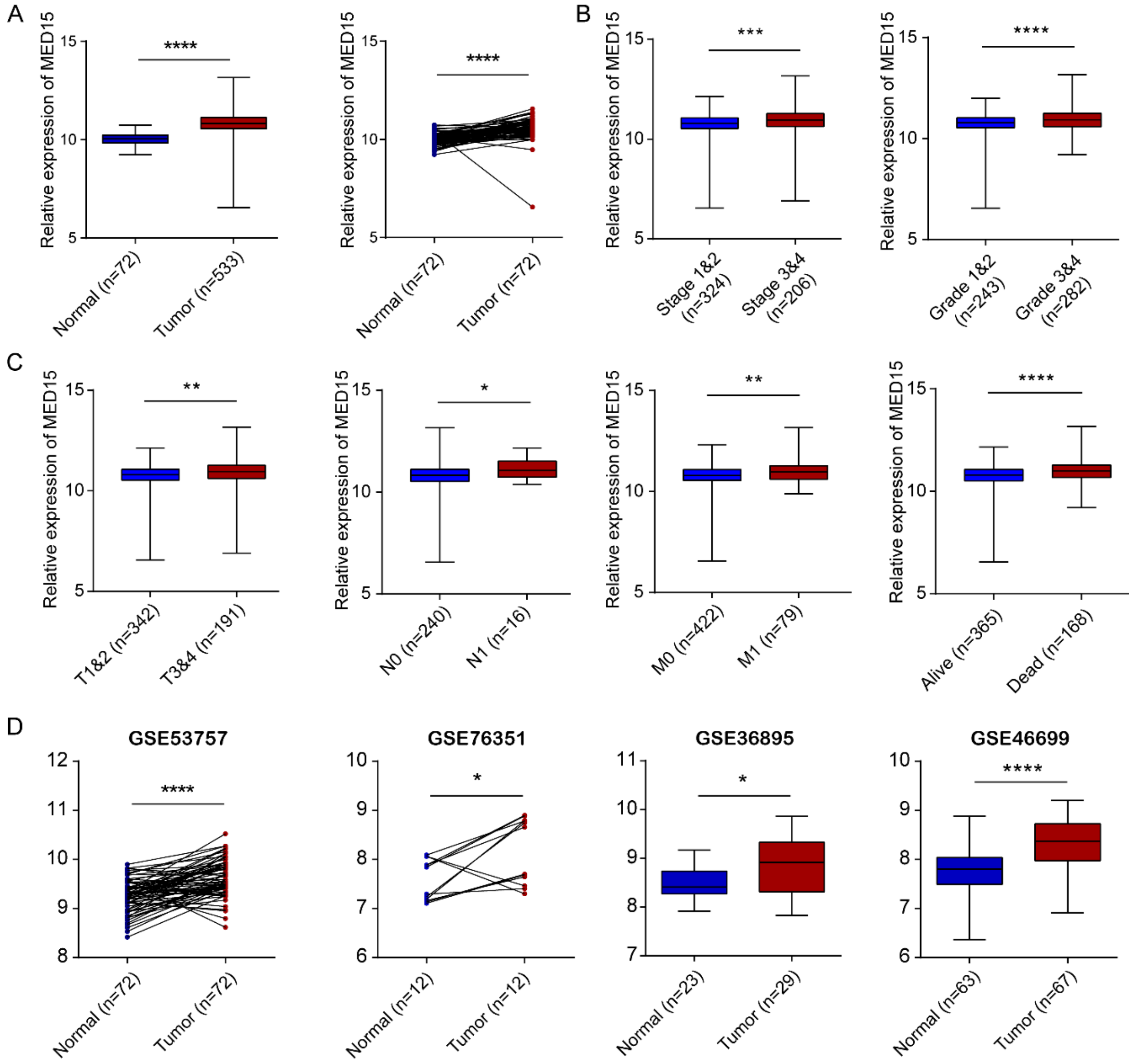

Fig. S1. MED15 was upregulated in ccRCC and positively associated with clinicopathologic features in publicly available datasets. (A) MED15 was upregulated in ccRCC samples in the TCGA dataset. (B-C) The expression of MED15 was positively associated with clinicopathologic features in the TCGA dataset. (D) MED15 was upregulated in ccRCC in four datasets from the GEO database. The results are expressed as the mean ± standard deviation (SD). * indicates P < 0.05; ** indicates P < 0.01; *** indicates P < 0.001; **** indicates P < 0.0001.

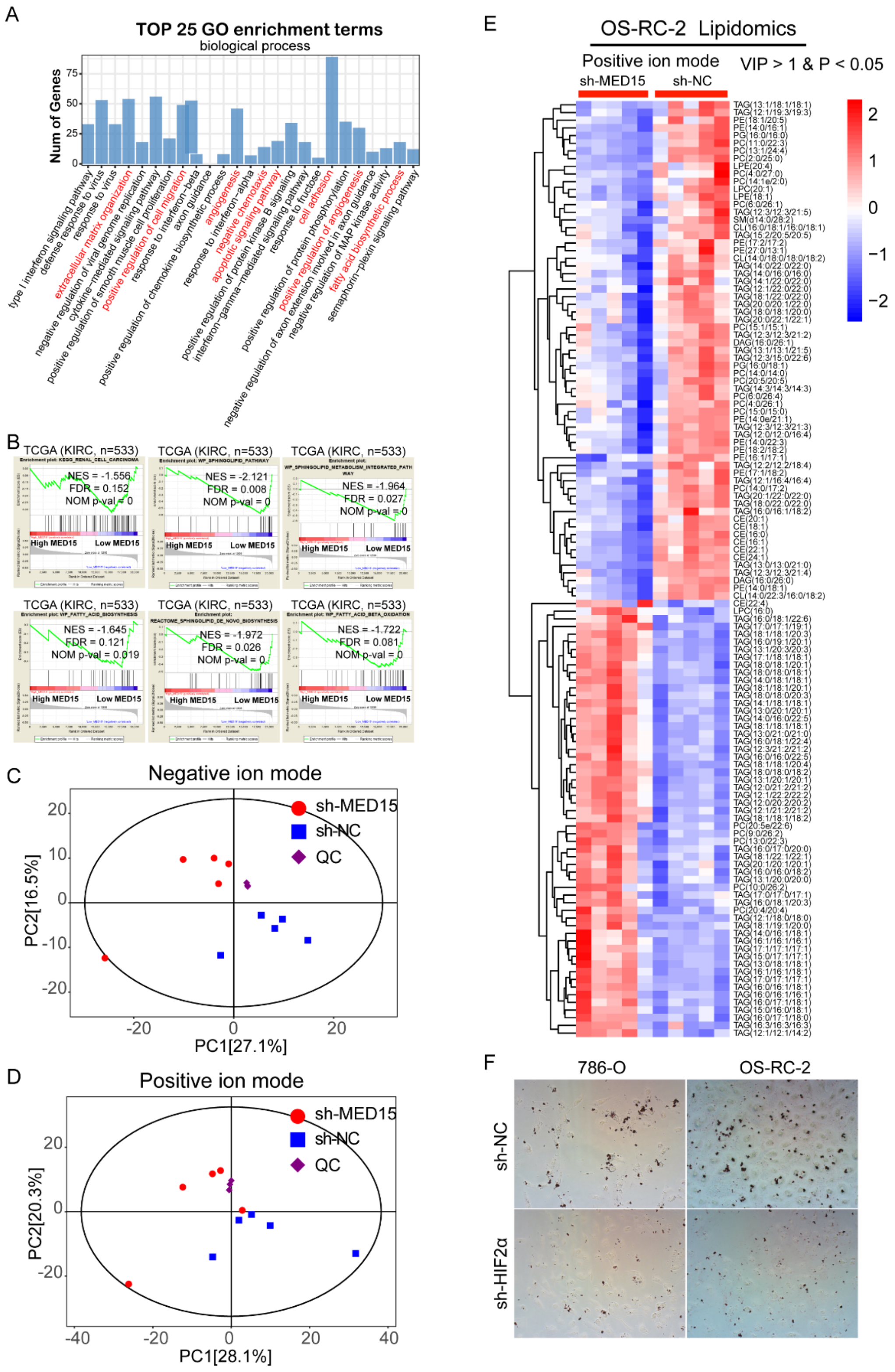

Fig. S2. MED15 promoted tumor progression and lipid accumulation in ccRCC. (A) The top 25 enriched biological process terms from the Gene Ontology (GO) enrichment analysis. (B) The enriched lipid accumulation-related terms from the gene set enrichment analysis (GSEA) of TCGA data. Principal component analysis of all lipid expression profiles detected for cells with or without MED15

knockdown in negative ion mode (C) and positive ion mode (D). (E) The differentially expressed lipid components in positive ion mode were detected by LC/MS lipidomic assay in OS-RC-2 cells with or without MED15 knockdown (n = 5). (F) Oil Red O staining in RCC cells with HIF-2α knockdown.

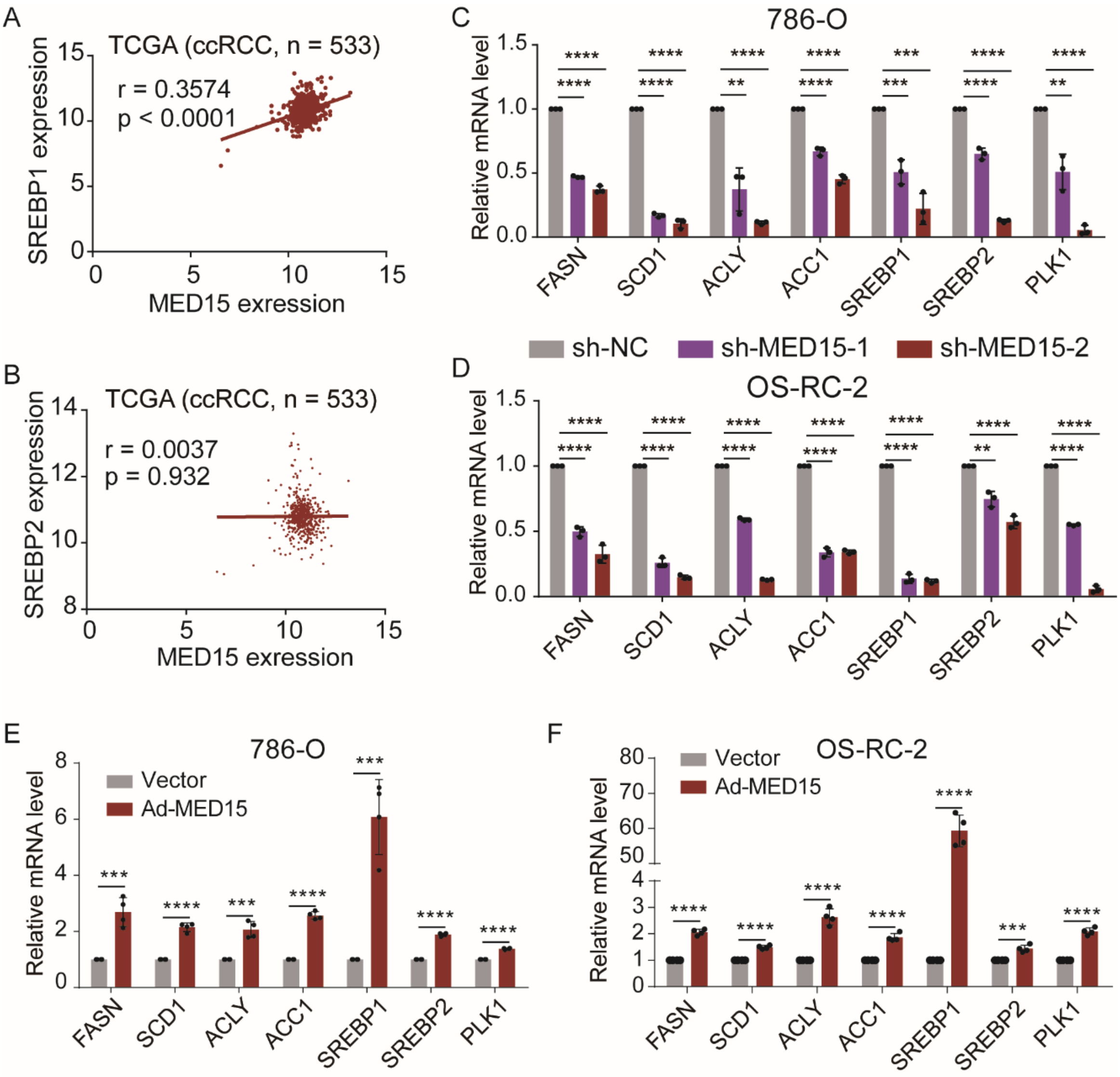

Fig. S3. MED15 promoted the activation of the SREBP pathway. (A-B) Correlations between MED15 expression and SREBP1 and SREBP2 expression. (C-D) qRT–PCR showed decreased protein levels of SREBP1, SREBP2, PLK1 and SREBP-dependent lipid biosynthesis enzymes (FASN, ACC1, ACLY, and SCD1) in RCC cells with MED15 knockdown (n = 3). (E-F) qRT–PCR showed increased protein levels of SREBP1, SREBP2, PLK1 and SREBP-dependent lipid biosynthesis enzymes (FASN, ACC1, ACLY, and SCD1) in RCC cells overexpressing MED15 (n = 3). The results are expressed as the mean ± standard deviation (SD). ** indicates $P < 0.01$; *** indicates $P < 0.001$; **** indicates $P < 0.0001$.

Table S1. Association between MED15 mRNA expression and clinicopathological characteristics of patients with clear cell renal cell carcinoma.

Variables	Number	MED15 mRNA expression		*P* value
		High (n = 267)	Low (n = 266)	
Age (years)				< 0.001
≤ 60	264 (49.5)	145 (54.3)	119 (44.7)	
> 60	269 (50.5)	122 (45.7)	147 (55.3)	
Gender				< 0.001
Male	345 (64.7)	176 (65.9)	169 (63.5)	
Female	188 (35.3)	91 (34.1)	97 (36.5)	
Stage				0.017
I	267 (50.1)	117 (43.8)	150 (56.4)	
II	57 (10.7)	27 (10.1)	30 (11.3)	
III	123 (23.1)	70 (26.2)	53 (19.9)	
IV	83 (15.6)	51 (19.1)	32 (12.0)	
Unkonwn	3 (0.5)	2 (0.8)	1 (0.4)	
Grade				< 0.001
G1	14 (2.6)	10 (3.7)	4 (1.4)	
G2	229 (43.0)	95 (35.6)	134 (50.4)	
G3	206 (38.6)	109 (40.8)	97 (36.5)	
G4	76 (14.3)	51 (19.1)	25 (9.4)	
Unkonwn	8 (1.5)	2 (0.8)	6 (2.3)	
T stage				0.006
T1	273 (51.2)	119 (44.6)	154 (57.9)	
T2	69 (12.9)	34 (12.7)	35 (13.2)	
T3	180 (33.8)	106 (39.7)	74 (27.8)	
T4	11 (2.1)	8 (3.0)	3 (1.1)	
N stage				0.448
N0/Nx	517 (97.0)	257 (96.3)	260 (97.7)	
N1	16 (3.0)	10 (3.7)	6 (2.3)	
M stage				0.051
M0/Mx	454 (85.2)	219 (82.0)	235 (88.3)	
M1	79 (14.8)	48 (18.0)	31 (11.7)	
Status				0.001
Alive	365 (68.5)	165 (61.8)	200 (75.2)	
Dead	168 (31.5)	102 (38.2)	66 (24.8)	

Table S2. Univariate and multivariate analyses of MED15 mRNA level for overall survival of patients.

**Variables**	**Univariate analysis**			**Multivariate analysis**		
	**HR**	**95% CI**	***P* value**	**HR**	**95% CI**	***P* value**
Overall survival (n = 517)						
Age (years)	1.719	1.255-2.352	< 0.001	1.651	1.203-2.265	0.002
≤60 (n = 260)						
>60 (n = 257)						
Gender	0.960	0.699-1.320	0.804			
Female (n = 180)						
Male (n = 337)						
T stage	1.987	1.680-2.350	< 0.001	1.319	1.070-1.626	0.009
T1 or T2 (n = 330)						
T3 or T4 (n = 187)						
N stage	3.545	1.866-6.732	< 0.001	1.464	0.743-2.883	0.271
N0 or NX (n = 503)						
N1 (n = 14)						
M stage	4.436	3.226-6.100	< 0.001	2.332	1.583-3.434	< 0.001
M0 or MX (n = 441)						
M1 (n = 76)						
Grade	2.322	1.881-2.866	< 0.001	1.474	1.161-1.871	0.001
G1 or G2 (n = 239)						
G3 or G4 (n = 278)						
MED15	1.793	1.308-2.457	< 0.001	1.518	1.098-2.098	0.011
Low (n = 258)						
High (n = 259)						

HR: Hazard ratio. CI: Confidence interval.

Multivariate models are adjusted for T (tumor), N (lymph node), M (metastasis) classification, age, stage, grade and MED15 expression level.

Table S3. Univariate and multivariate analyses of MED15 mRNA level for disease free survival (DFS) of ccRCC patients.

**Variables**	**Univariate analysis**			**Multivariate analysis**		
	**HR**	**95% CI**	***P* value**	**HR**	**95% CI**	***P* value**
DFS (n = 422)						
Age (years)	1.366	0.959-1.945	0.084			
≤60 (n = 230)						
>60 (n = 192)						
Gender	1.429	0.962-2.123	0.077			
Female (n = 142)						
Male (n = 280)						
T stage	4.527	3.134-6.539	< 0.001	2.056	1.349-3.134	0.001
T1 or T2 (n = 283)						
T3 or T4 (n = 139)						
N stage	5.955	2.990-11.861	< 0.001	3.236	1.600-6.545	0.001
N0 or NX (n = 410)						
N1 (n = 12)						
M stage	8.537	5.882-12.398	< 0.001	5.271	3.467-8.012	< 0.001
M0 or MX (n = 371)						
M1 (n = 51)						
Grade	1.832	1.491-2.252	< 0.001	1.557	1.258-1.927	< 0.001
G1 or G2 (n = 208)						
G3 or G4 (n = 214)						
MED15	1.537	1.074-2.200	0.019	1.457	1.016-2.090	0.041
Low (n = 211)						
High (n = 211)						

HR: Hazard ratio. CI: Confidence interval.

Multivariate models are adjusted for T (tumor), N (lymph node), M (metastasis) classification, age, stage, grade and MED15 expression level.

Table S4: The results of immunohistochemical staining for MED15 and clinical characteristics in tissue microarray.

HKidE180Su02	Tissue type	T	Status	Survival time (month)	MED15 expression
A01	Tumor	T1	alive	90	weak
A02	Normal				weak
A03	Tumor	T1	dead	14	moderate
A04	Normal				moderate
A05	Tumor	T1	alive	90	weak
A06	Normal				moderate
A07	Tumor	T1	alive	90	moderate
A08	Normal				moderate
A09	Tumor	T1	dead	24	positive
A10	Normal				weak
A11	Tumor	T1	alive	89	moderate
A12	Normal				moderate
A13	Tumor	T1	alive	88	moderate
A14	Normal				moderate
A15	Tumor	T1	alive	88	moderate
A16	Normal				weak
A17	Tumor	T1	alive	88	moderate
A18	Normal				weak
B01	Tumor	T1	alive	88	weak
B02	Normal				weak
B03	Tumor	T1	alive	88	weak
B04	Normal				weak
B05	Tumor	T1	alive	88	moderate
B06	Normal				moderate
B07	Tumor	T3	alive	88	positive
B08	Normal				moderate
B09	Tumor	T1	alive	88	moderate
B10	Normal				moderate
B11	Tumor	T1	alive	87	moderate
B12	Normal				moderate
B13	Tumor	T2	dead	38	positive
B14	Normal				positive
B15	Tumor	T1	alive	87	moderate
B16	Normal				moderate
B17	Tumor	T1	alive	87	moderate
B18	Normal				weak
C01	Tumor	T1	alive	87	weak
C02	Normal				weak
C03	Tumor	T2	alive	87	positive

C04	Normal				moderate
C05	Tumor	T1	alive	86	moderate
C06	Normal				moderate
C07	Tumor	T1	alive	86	moderate
C08	Normal				moderate
C09	Tumor	T1	alive	86	moderate
C10	Normal				moderate
C11	Tumor	T1	alive	86	positive
C12	Normal				moderate
C13	Tumor	T1	alive	86	moderate
C14	Normal				moderate
C15	Tumor	T1	alive	86	moderate
C16	Normal				positive
C17	Tumor	T1	alive	86	moderate
C18	Normal				weak
D01	Tumor	T1	alive	86	moderate
D02	Normal				moderate
D03	Tumor	T1	alive	86	moderate
D04	Normal				moderate
D05	Tumor	T2	dead	31	positive
D06	Normal				moderate
D07	Tumor	T1	alive	85	positive
D08	Tumor	T1	dead	10	positive
D09	Tumor	T2	alive	85	positive
D10	Tumor	T1	alive	85	positive
D11	Tumor	T1	alive	85	positive
D12	Tumor	T1	alive	85	moderate
D13	Tumor	T1	alive	85	moderate
D14	Tumor	T3	dead	41	positive
D15	Tumor	T1	alive	85	moderate
D16	Tumor	T1	alive	85	weak
D17	Tumor	T1	alive	85	moderate
D18	Tumor	T3	dead	52	strong
E01	Tumor	T2	dead	15	positive
E02	Tumor	T1	alive	84	moderate
E03	Tumor	T3	alive	84	positive
E04	Tumor	T1	alive	84	moderate
E05	Tumor	T2	alive	84	positive
E06	Tumor	T1	alive	84	moderate
E07	Tumor	T2	alive	84	positive
E08	Tumor	T1	alive	83	moderate
E09	Tumor	T1	alive	83	moderate
E10	Tumor	T2	dead	46	positive
E11	Tumor	T1	dead	51	positive

E12	Tumor	T1	alive	81	moderate
E13	Tumor	T1	alive	81	strong
E14	Tumor	T1	alive	81	positive
E15	Tumor	T1	alive	81	moderate
E16	Tumor	T1	alive	80	moderate
E17	Tumor	T1	alive	80	moderate
E18	Tumor	T1	dead	51	positive
F01	Tumor	T1	alive	80	positive
F02	Tumor	T1	dead	40	weak
F03	Tumor	T1	alive	79	moderate
F04	Tumor	T1	alive	79	moderate
F05	Tumor	T1	alive	79	moderate
F06	Tumor	T1	alive	78	strong
F07	Tumor	T1	alive	78	positive
F08	Tumor	T3	dead	22	strong
F09	Tumor	T2	alive	77	strong
F10	Tumor	T1	alive	77	positive
F11	Tumor	T1	alive	77	moderate
F12	Tumor	T3	dead	4	positive
F13	Tumor	T1	alive	77	positive
F14	Tumor	T1	alive	76	moderate
F15	Tumor	T1	alive	76	positive
F16	Tumor	T1	alive	76	positive
F17	Tumor	T2	dead	38	positive
F18	Tumor	T3	dead	7	strong
G01	Tumor	T3	dead	7	positive
G02	Tumor	T1	alive	76	positive
G03	Tumor	T1	alive	75	positive
G04	Tumor	T1	alive	75	moderate
G05	Tumor	T1	alive	75	positive
G06	Tumor	T1	alive	75	positive
G07	Tumor	T1	alive	74	positive
G08	Tumor	T1	alive	74	positive
G09	Tumor	T1	alive	74	positive
G10	Tumor	T1	dead	21	positive
G11	Tumor	T1	alive	74	positive
G12	Tumor	T1	alive	74	strong
G13	Tumor	T1	alive	74	positive
G14	Tumor	T2	dead	4	positive
G15	Tumor	T3	dead	5	strong
G16	Tumor	T1	alive	73	positive
G17	Tumor	T2	dead	14	positive
G18	Tumor	T1	alive	72	moderate
H01	Tumor	T1	alive	72	moderate

H02	Tumor	T1	alive	72	weak
H03	Tumor	T3	alive	72	strong
H04	Tumor	T1	alive	72	positive
H05	Tumor	T1	alive	72	strong
H06	Tumor	T1	alive	72	positive
H07	Tumor	T1	alive	72	positive
H08	Tumor	T1	alive	72	positive
H09	Tumor	T1	alive	71	strong
H10	Tumor	T1	alive	71	positive
H11	Tumor	T1	alive	71	positive
H12	Tumor	T1	alive	71	positive
H13	Tumor	T1	alive	71	strong
H14	Tumor	T1	alive	71	positive
H15	Tumor	T1	alive	71	positive
H16	Tumor	T1	alive	71	positive
H17	Tumor	T1	alive	71	positive
H18	Tumor	T1	alive	70	strong
I01	Tumor	T1	dead	43	positive
I02	Tumor	T3	alive	70	positive
I03	Tumor	T1	alive	70	strong
I04	Tumor	T1	alive	70	positive
I05	Tumor	T1	alive	70	moderate
I06	Tumor	T1	alive	69	positive
I07	Tumor	T1	alive	69	strong
I08	Tumor	T1	alive	69	moderate
I09	Tumor	T2	dead	33	strong
I10	Tumor	T1	alive	69	positive
I11	Tumor	T1	alive	69	positive
I12	Tumor	T1	alive	69	positive
I13	Tumor	T1	alive	69	positive
I14	Tumor	T2	alive	68	positive
I15	Tumor	T1	alive	68	positive
I16	Tumor	T2	alive	68	weak
I17	Tumor	T1	alive	68	positive
I18	Tumor	T1	alive	68	positive
J01	Tumor	T1	dead	41	positive
J02	Tumor	T1	dead	66	positive
J03	Tumor	T1	alive	67	positive
J04	Tumor	T1	alive	67	positive
J05	Tumor	T1	alive	67	positive
J06	Tumor	T1	alive	67	positive
J07	Tumor	T1	dead	49	positive
J08	Tumor	T1	alive	67	positive
J09	Tumor	T1	alive	67	positive

J10	Tumor	T1	alive	67	strong
J11	Tumor	T2	dead	57	positive
J12	Tumor	T1	dead	7	positive
J13	Tumor	T1	alive	66	positive
J14	Tumor	T1	alive	66	positive
J15	Tumor	T1	alive	66	strong
J16	Tumor	T1	alive	65	positive
J17	Tumor	T1	alive	65	positive
J18	Tumor	T2	alive	65	positive

Table S5: Lentivirus construction information.

Name	Gene ID	Genebank	Vector information
HIF2α-RNAi	2034	NM_001430	pHBLV-U6-MCS-CMV-ZsGreen-PGK-PURO
MED15-RNAi	51586	NM_001293234	hU6-MCS-Ubiquitin-EGFP-IRES-puromycin, GV248
MED15	51586	NM_015889	CMV-MCS-3FLAG-EF1a-mCherry-T2A-puromycin, GV661
HIF2α	2034	NM_001430	Ubi-MCS-3FLAG-SV40-EGFP-IRES-puromycin, GV358

Table S7: Primers for qRT-PCR analysis.

Symbol	Forward primer (5'--3')	Reverse primer (5'--3')
HIF-2α	CACCAAGGGTCAGGTAGTAAG	CACCAAGGGTCAGGTAGTAAG
MED15	ATGGACGTTTCCGGGCAAG	GCATCCTCGATTTGACTGACCA
SREBP1	ACAGTGACTTCCCTGGCCTAT	GCATGGACGGGTACATCTTCAA
SREBP2	AACGGTCATTCACCCAGGTC	GGCTGAAGAATAGGAGTTGCC
FASN	AAGGACCTGTCTAGGTTTGATGC	TGGCTTCATAGGTGACTTCCA
SCD1	GCCCCTCTACTTGGAAGACGA	AAGTGATCCCATACAGGGCTC
ACC1	CATGCGGTCTATCCGTAGGTG	GTGTGACCATGACAACGAATCT
ACLY	ATCGGTTCAAGTATGCTCGGG	GACCAAGTTTTCCACGACGTT
PLK1	CACCAGCACGTCGTAGGATTC	CCGTAGGTAGTATCGGGCCTC
GAPDH	GGGAGCCAAAAGGGTCAT	GAGTCCTTCCACGATACCAA

Table S8: List of primers for chromatin immunoprecipitation assay.

Symbol	Forward primer (5'--3')	Reverse primer (5'--3')
Negative control	AACCCCGGTCAAGATGATCACTC	TTCTCCTGCCTCAGCCTCCCAAG
Site 1	ACTGGAAGAGGGGCGTGTGT	GAAATGGAGTCTCGCGCACAG
Site 2	TGGCTGCTGGTCAAAAGGGAA	TGGACTCTCATCACCGCTTCA
Site 3	CCCATAACCCCTCATCCCACC	AGACATGGGCTTTCCCTTTTG
Site 4	GGTGGGGTACGCTTGTAATC	GGGATAGAATTTAGCCCTTGT
VEGF	CTTTGGGTTTTGCCAGACTCC	GGAAGTGTCCAGGGATGCT

Table S9: The specific sequences of truncated plasmid.

Site 1--(MED15)
CGTTGCACTGGCACACAGTAGGTACCCACACATATTTGATAAATAAACACTCTCTCCCT
AGCATTACTATAGATAGCTGGCAGCTTGGCCTGGCATCCCGGAGGGGTCTGGATCTCAG
GGACTAGATAAGGCCAGCACACTGAAGAGTTGCAGAAAATGCAGGCGGTCAGGCGCAG
GTCTGCTGCGGGAGCACCTGCCGTGTCCGACAGCTGCACTGGAAGAGGGGCGTGTGTCT
CCCAGCCGGGCGGAACCGAGACCGCTTCGTCCCCGGCGGGGAACTTCATTTCCCACAAG
CCTGTGCGCGAGACTCCATTTCCCACAAGCCCTCCTTCACTTCCGGCTCGGCCGAGGCA

Site 2+ Site 1--(MED15)
CAAAAGGGAAAGCCCATGTCTAAAAATTAATTAACGTGGTTTAGGGGCCTCACATTCCC
AACCCCCAAATACAGCCATTGTGAGATGTGAGATTCAAAAAATCGAAGAGTGAAGCGG
TGATGAGAGTCCATCTGGTCGCTCTTCGGATGCGGCGCTAGCAGGCAGTTGCATGAAAG
CAGATCCCAGATCACGTTGCACTGGCACACAGTAGGTACCCACACATATTTGATAAATA
AACACTCTCTCCCTAGCATTACTATAGATAGCTGGCAGCTTGGCCTGGCATCCCGGAGG
GGTCTGGATCTCAGGGACTAGATAAGGCCAGCACACTGAAGAGTTGCAGAAAATGCAG
GCGGTCAGGCGCAGGTCTGCTGCGGGAGCACCTGCCGTGTCCGACAGCTGCACTGGAA
GAGGGGCGTGTGTCTCCCAGCCGGGCGGAACCGAGACCGCTTCGTCCCCGGCGGGGAA
CTTCATTTCCCACAAGCCTGTGCGCGAGACTCCATTTCCCACAAGCCCTCCTTCACTTCC
GGCTCGGCCGAGGCA

Site 3+ Site 2+ Site 1--(MED15)
CTCCCACCCACTCTCTACCACCTTCAACCTCCTGTCTGGGGCACAGTAACAAGGGGTGT
AAGGGGTGTACAGTGCCTAGCAGGGGCCTGGCACTTAGGCTTCAGTAGGTGCCAGTCCA
CAGTCTCCTGAGGCCCATGTTGGTTGGTGTCCACCTGGTGCTTATTGACCACTTGGGCTC
CAGATTTTCCTCACGTCACCTCCCTCCCCAGGGTAGCAGATAAAGGCCCCATAGTTCCA
AGGAGGCAGAACTTTTACATTCCTAAAAGCATTCTCAGGCCCCCATAACCCCTCATCCC
ACCTTCAGCTCGCTCACCTTCACCTATCAAATTCGTTTGACATCTGGCCTCCAGCAACTA
GCTAGGCTGAGTTCTCCATGAAGGGGACGTGGCTGCTGGTCAAAAGGGAAAGCCCATG
TCTAAAAATTAATTAACGTGGTTTAGGGGCCTCACATTCCCAACCCCCAAATACAGCCA
TTGTGAGATGTGAGATTCAAAAAATCGAAGAGTGAAGCGGTGATGAGAGTCCATCTGGT
CGCTCTTCGGATGCGGCGCTAGCAGGCAGTTGCATGAAAGCAGATCCCAGATCACGTTG
CACTGGCACACAGTAGGTACCCACACATATTTGATAAATAAACACTCTCTCCCTAGCAT
TACTATAGATAGCTGGCAGCTTGGCCTGGCATCCCGGAGGGGTCTGGATCTCAGGGACT
AGATAAGGCCAGCACACTGAAGAGTTGCAGAAAATGCAGGCGGTCAGGCGCAGGTCTG
CTGCGGGAGCACCTGCCGTGTCCGACAGCTGCACTGGAAGAGGGGCGTGTGTCTCCCAG
CCGGGCGGAACCGAGACCGCTTCGTCCCCGGCGGGGAACTTCATTTCCCACAAGCCTGT
GCGCGAGACTCCATTTCCCACAAGCCCTCCTTCACTTCCGGCTCGGCCGAGGCA

Site 4+ Site 3+ Site 2+ Site 1--(MED15)
CAAAAATTAGCCAGGCATGGTGGGGTACGCTTGTAATCCCAGCTACTGGGGAAGCTGA
GGTGGGAGAATTGCGTGAACCCGGGAGACAGAGGTTGCAGTGAGCCGAGATGGCACCA
CTGCACTCCAGCCTGGCCAACAAGGGCTAAATTCTATCCCCCCCCCCTCAAAAAAAACA

ATAACGATTTTGGGAGTATTAGAGCAGAAAACGTTTGAAAACATGAACAGCTCTCACAT
CAACACAAGACATGAAACTCTGTTGGCAAGAGAGGCCTCTTTGGGGAGAGTGCTCACTC
CTCTCCCACCCACTCTCTACCACCTTCAACCTCCTGTCTGGGGCACAGTAACAAGGGGTG
TAAGGGGTGTACAGTGCCTAGCAGGGGCCTGGCACTTAGGCTTCAGTAGGTGCCAGTCC
ACAGTCTCCTGAGGCCCATGTTGGTTGGTGTCCACCTGGTGCTTATTGACCACTTGGGCT
CCAGATTTTCCTCACGTCACCTCCCTCCCCAGGGTAGCAGATAAAGGCCCCATAGTTCC
AAGGAGGCAGAACTTTTACATTCCTAAAAGCATTCTCAGGCCCCCATAACCCCTCATCC
CACCTTCAGCTCGCTCACCTTCACCTATCAAATTCGTTTGACATCTGGCCTCCAGCAACT
AGCTAGGCTGAGTTCTCCATGAAGGGGACGTGGCTGCTGGTCAAAAGGGAAAGCCCAT
GTCTAAAAATTAATTAACGTGGTTTAGGGGCCTCACATTCCCAACCCCCAAATACAGCC
ATTGTGAGATGTGAGATTCAAAAAATCGAAGAGTGAAGCGGTGATGAGAGTCCATCTG
GTCGCTCTTCGGATGCGGCGCTAGCAGGCAGTTGCATGAAAGCAGATCCCAGATCACGT
TGCACTGGCACACAGTAGGTACCCACACATATTTGATAAATAAACACTCTCTCCCTAGC
ATTACTATAGATAGCTGGCAGCTTGGCCTGGCATCCCGGAGGGGTCTGGATCTCAGGGA
CTAGATAAGGCCAGCACACTGAAGAGTTGCAGAAAATGCAGGCGGTCAGGCGCAGGTC
TGCTGCGGGAGCACCTGCCGTGTCCGACAGCTGCACTGGAAGAGGGGCGTGTGTCTCCC
AGCCGGGCGGAACCGAGACCGCTTCGTCCCCGGCGGGGAACTTCATTTCCCACAAGCCT
GTGCGCGAGACTCCATTTCCCACAAGCCCTCCTTCACTTCCGGCTCGGCCGAGGCA

Table S10. Publicly-available datasets used in the study.

Dataset	Samples	Data Array	Sample Description	Reference
TCGA	605	RNA-seq	72 normal samples and 533 ccRCC samples	https://xenabrowser.net/
GSE53757	144	Affymetrix Human Genome U133 Plus 2.0 Array	72 normal samples and 72 ccRCC samples	von Roemeling CA, et al. Cancer Res. 2014;74(17):4796-810
GSE76351	24	Affymetrix Human Gene 1.1 ST Array	12 normal samples and 12 ccRCC samples	None
GSE36895	52	Affymetrix Human Genome U133 Plus 2.0 Array	23 normal samples and 29 ccRCC samples	Peña-Llopis S, et al. Nat Genet. 2012;44(7):751-9
GSE46699	130	Affymetrix Human Genome U133 Plus 2.0 Array	63 normal samples and 67 ccRCC samples	Eckel-Passow JE, et al. Carcinogenesis. 2014; 35(4):822-7

Abbreviations: GSE, data set accession ID in Gene Expression Omnibus; TCGA, The Cancer Genome Atlas; ccRCC, clear cell renal cell carcinoma.

Table S11: The list of lipid metabolism-related gene set.

NFYA	GDE1	MBTPS2	AGPAT4	THRAP3	CTSA	SLC44A1	PTGS2
CYP51A1	NR1H4	AC003665.1	PNPLA6	GALC	FAR2	GBA2	MGLL
MTMR7	AGK	ELOVL5	CYP46A1	TBXAS1	ME1	CHAT	HACD3
NDUFAB1	ALDH3B1	ALOX5	PI4K2B	ACAA1	NFYC	CSNK2A2	ACAT1
ACSM3	ARSD	DPEP1	INPP4A	ARSF	ELOVL1	OSBPL3	PLD1
CREBBP	PNPLA4	AGPS	MED17	DGAT2	IDI1	PRKACA	ACACB
TSPOAP1	MED24	CYP24A1	HEXB	SPHK2	PLPP1	ALDH3A2	ACER3
PON1	OSBPL5	STARD3NL	PIK3CB	MED29	ACSL4	SREBF1	PIK3C3
CROT	ABHD5	ALAS1	PLEKHA5	AHRR	RORA	ACADVL	SYNJ2
ABCB4	PIK3C2A	NR1H3	MSMO1	MTMR1	PLA2G10	ABCB11	OSBPL6
PCYT1B	INSIG2	PPARD	TGS1	INPP5E	MTMR14	PPP1CA	ELOVL2
GLA	MED1	CCNC	FDX1	ANKRD1	PLB1	CYP7B1	HSD17B11
GDPD3	GPCPD1	MED23	CYP19A1	CYP17A1	LIPH	DHCR7	TXNRD1
VAC14	NR1D1	ACOT13	SLC44A5	HSD17B12	SGMS2	LCLAT1	AKR1C4
SMPD3	SGPP1	FIG4	SELENOI	FADS1	BDH2	ACSM6	NCOA6
PLA2G15	PLAAT1	FAM120B	HADHB	TM7SF2	ETNPPL	ESRRA	ECI2
FA2H	PLA2G5	HMGCS1	CYP1B1	MTMR12	HPGD	ARV1	OXCT2
MLYCD	SIN3B	HMGCR	CYP2C9	PIP4K2A	ELOVL7	PHOSPHO1	GK
ABCC1	ECHS1	COL4A3BP	CYP2C8	SEC24D	STARD4	STARD6	INPP5F
UBE2I	SRD5A3	ARSB	CH25H	THRSP	ARSK	UGT8	SREBF2
CDIPT	ORMDL1	SEC24A	GPAT3	MMAA	ENPP6	PTDSS2	SGMS1
ACSBG1	SUMF2	EHHADH	HADH	AKR1C2	ACSL6	PCCA	HSD3B1
DECR1	PLD2	PCCB	SEC24B	ACSL1	FABP7	MED16	HSD3B2
SQLE	SLC44A2	BCHE	PIK3C2G	OLAH	FABP5	GPX2	NEU4
MTMR9	SLC27A1	SCAP	ETNK1	GPD1L	MED30	SPHK1	AWAT1
ASAH1	ACSBG2	PIKFYVE	GLIPR1	SAR1B	ABCA1	CIDEA	HSD17B8
LHB	HELZ2	POMC	GLTP	GGPS1	PRKACG	ACBD7	RXRB
MED25	CYP2E1	GPD2	MTMR6	MED21	MID1IP1	HSD11B2	AGPAT1
MED26	PNPLA7	ACADL	CERS5	ACOXL	SPTSSA	LPCAT4	SLC44A4
PLD3	OSBPL2	PECR	ESYT1	PLA2R1	INPPL1	PLAAT3	NEU1
PIAS4	MED18	NEU2	FITM1	LPCAT1	FAAH2	ACSF3	CSNK2B
PRKD2	HSD17B3	FHL2	PTGR2	GDPD1	PIP4P1	RUFY1	FABP9
PLIN3	PPT1	PRKD3	SLC27A2	CERS3	CYP2C19	SEC24C	CPT1B
SULT2A1	HACL1	PLEKHA3	NCOA2	CYP2U1	HACD1	ACER2	ARSH
PLA2G4C	NR1H2	DHCR24	CYP11A1	CDK19	SGPL1	CHD9	ACOT6
PLEKHA4	ACLY	SCP2	CYP1A1	AGPAT5	SMPD1	ACOT4	TECRL
PIK3R2	STARD3	MECR	CYP1A2	PI4K2A	MOGAT2	TBL1XR1	PLPP6
PIK3CG	PRKAB2	PLA2G4A	AC092724.1	DBI	PLD4	GBA	HACD2
PON3	PPARG	HAO2	MBTPS1	PPARGC1B	PLIN1	PNPLA2	SACM1L
PON2	HSD17B7	GNPAT	MED9	MED7	PEX11A	SLC25A20	LTC4S
CAV1	RAN	ACADM	NCOR1	PTDSS1	PIP4K2C	CPNE7	HEXA
PLEKHA8	INPP5K	PLA2G2D	DPEP3	MED19	MTMR10	CYP11B2	PPP1CB

MOGAT3	GPS2	HMGCL	PCTP	SAMD8	ACSF2	ALOXE3	NUDT19
NRF1	ALDH3B2	PIK3R3	ARSG	CPT2	DPEP2	ALOX12B	CPNE1
AHR	LPIN3	FAAH	OSBPL1A	ARSE	ACAA2	ALOX15B	STARD10
PRKAG2	TPTE2	HSD11B1	PIK3R5	PRXL2B	GPX4	PLD6	PPT2
PTGR1	CDK8	OSBPL9	PLPP2	ABHD3	MVD	TMEM86B	CERS1
PIP5K1B	ALOX5AP	ESYT2	CARM1	ESYT3	GPD1	MED14	CPTP
PTGDS	PEMT	APOA1	PRKACB	GDPD5	ACER1	CYP8B1	AKR1B15
PHYH	PIK3C2B	ELOVL4	CYP4B1	GPAT4	ANGPTL4	MIGA1	GK3P
CUBN	CSNK1G2	MED28	SLC44A3	PLA2G2F	CYP7A1	ARSJ	ACBD6
PLEKHA1	PLAAT4	RAB14	RGL1	APOA2	ECI1	PITPNB	CYP21A2
ACBD5	PLAAT2	KDSR	PI4KB	SYNJ1	PLAAT5	ACBD4	GPX1
MTMR4	MED10	ACOT2	PIP5K1A	CBR1	RAB4A	ENPP7	ACAD11
KPNB1	MORC2	ELOVL3	CERS2	PLA2G4D	ACOX2	TNFAIP8L3	UGT1A9
MED13	ARNTL	GPAM	ARNT	THEM4	DEGS2	ARSI	PISD
MED31	SBF2	ACAT2	SLC27A3	MED8	MFSD2A	DGAT2L6	PI4KA
HSD17B1	HSD17B4	NFYB	DEGS1	AGPAT3	GSTM4	ACOT1	AMACR
ALOX12	MED6	EPHX2	ARF1	LSS	PLA2G4F	PRKD1	DECR2
ABCC3	HMGCS2	PLBD1	MBOAT2	MED27	INPP5D	PLA2G6	PLA2G4B
DHRS7B	CEPT1	FABP3	LBR	FDPS	B3GALNT1	MED12	HTD2
INPP4B	ARF3	PIK3CA	ETNK2	CYP3A4	SIN3A	DGAT1	FDX2
PPARGC1A	GRHL1	OCRL	PLEKHA6	CYP11B1	PLEKHA2	INPP5J	TPTE
SC5D	LPIN1	AKR1D1	SUMF1	PCYT1A	AGPAT2	TNFAIP8L1	PIK3R6
OSBP	SLCO1B1	ACADS	RAB5A	BDH1	FASN	SP1	PIP4K2B
CPT1A	CYP2J2	MED13L	OSBPL10	FDXR	FABP6	PCYT2	SRD5A2
APOA5	FADS2	ACOT9	PLA1A	ACOX1	GLB1	CYP2R1	ACACA
PTPMT1	CLOCK	ORMDL2	SCD5	ALOX15	FABP4	PIP5K1C	SLCO1B3
PITPNM1	CD36	LPGAT1	GC	MED11	NPAS2	CYP4F2	MBOAT7
CHKA	PNPLA8	G0S2	FABP2	NEU3	HSD17B13	GPAT2	EBP
MVK	HILPDA	PLA2G12A	CBR4	CYP4A22	ELOVL6	PPP1CC	AWAT2
PTGES3	CGA	ACSL3	SRD5A1	ACOT11	PLA2G1B	RXRA	NSDHL
CYP27B1	BAAT	MOGAT1	PIK3R1	PLPP3	MTM1	INSIG1	STAR
ACSS3	SMPD4	B4GALNT1	ACAD10	LTA4H	CHPT1	VDR	LPCAT3
LIPE	HADHA	MTMR8	LIPI	CPNE6	ARNT2	MTMR3	CYP4F3
FDFT1	AKR1B1	TAZ	AKR1C3	SEC23A	ACOT12	PNPLA5	PPARA
LRP2	CPNE3	LPCAT2	THEM5	TRIB3	ALPI	PNPLA3	CYP4A11
AACS	DDHD2	CRLS1	PIK3R4	CSNK2A1	PMVK	SLC25A17	AKR1C1
SMARCD3	HSD17B2	SPTLC1	GK2	SCD	GLB1L	EP300	CIDEC
OXCT1	ACOX3	CERS4	NCOR2	HSD3B7	FABP1	PRKAA2	PIK3CD
SLC27A5	MTMR2	PITPNM2	SPTSSB	TECR	PPM1L	ACP6	HDAC3
STARD7	CRAT	OSBPL8	GM2A	MED15	CDS1	MAPKAPK2	PTEN
SLCO1A2	ACHE	PITPNM3	ACSL5	GGT5	PTPN13	SGPP2	CYP4F11
NCOA1	PGS1	PTGS1	FITM2	GGT1	ALB	HPGDS	CYP4F22
AGMO	CDS2	HSD17B14	SLC22A5	SLC25A1	IDI2	TNFAIP8L2	ORMDL3

PLA2G4E	ACOT8	ACOT7	FABP12	PLA2G3	PLIN2	NCOA3	TNFAIP8
PLA2G2A	VAPA	CERK	FAR1	CYP2D6	UGCG	VAPB	TNFRSF21
ASAH2	LPIN2	ABHD4	PSAP	SBF1	MED22	PTGIS	MMUT
SUMO2	STS	DDHD1	MBOAT1	CHKB	PTGES2	MCEE	HMGCLL1
PLA2G2E	TBL1X	SPTLC2	CERS6	MCAT	MIGA2	MED20	CYP39A1
MTF1	ABCD1	LGMN	SPTLC3	ARSA	PTGES	SLC10A2	TIAM2
HACD4	BMX	SLC10A1	STARD5	TSPO	CYP4F8	CYP27A1	SMPD2
MED4	AGT						
