# Supplementary material for: MED15 is upregulated by HIF-2α and promotes proliferation and metastasis in clear cell renal cell carcinoma via activation of SREBP-dependent fatty acid synthesis
Source: Cell Death Discov. 2024 Apr 22;10:188. doi: 10.1038/s41420-024-01944-1 (PMC11035615; doi:10.1038/s41420-024-01944-1)
Supplement: Supplementary file 3 — Original Western blot strips [file 41420_2024_1944_MOESM3_ESM.pdf]

Figure 1G

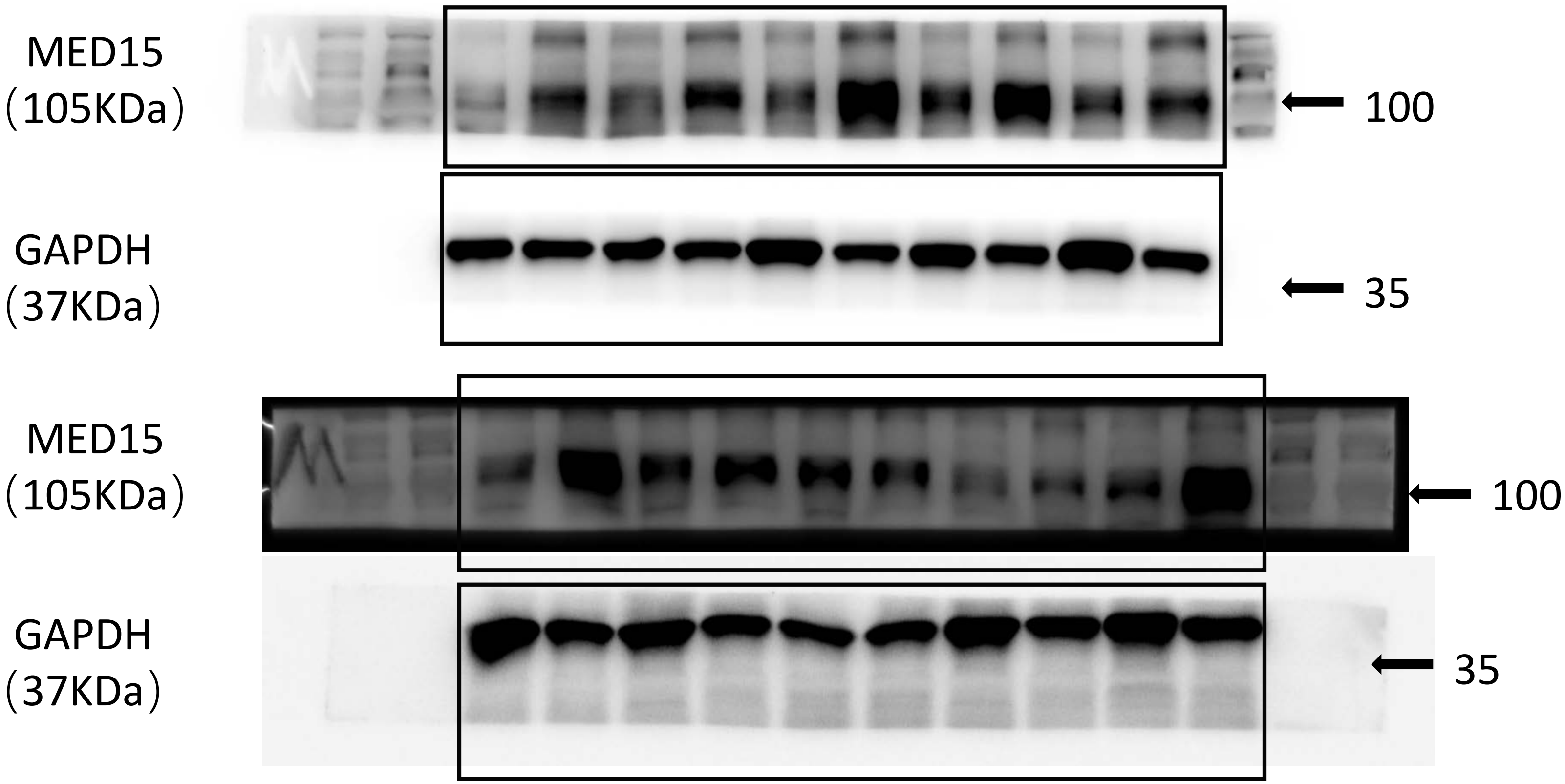

MED15
(105KDa)
100
GAPDH
(37KDa)
35
MED15
(105KDa)
100
GAPDH
(37KDa)
35

Figure 1I

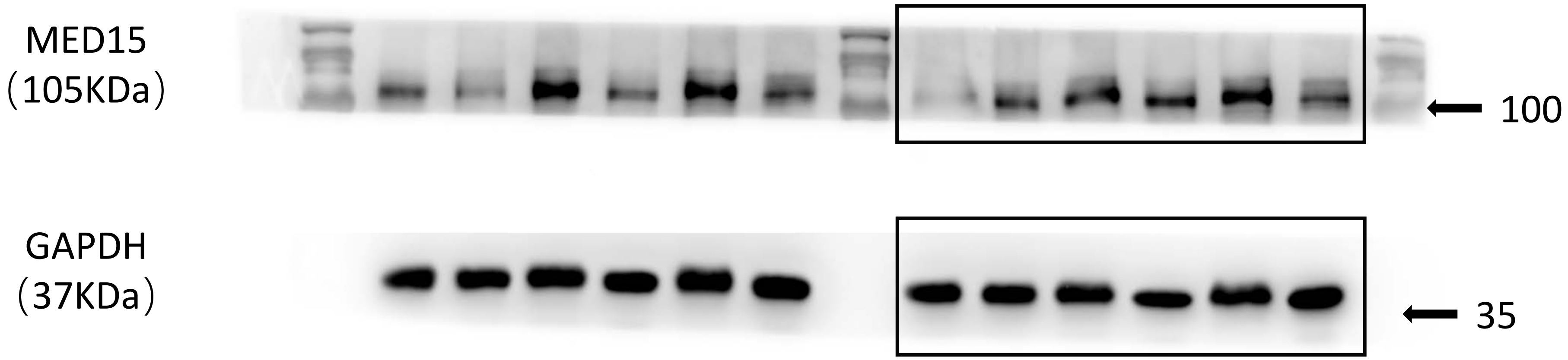

Figure 2A

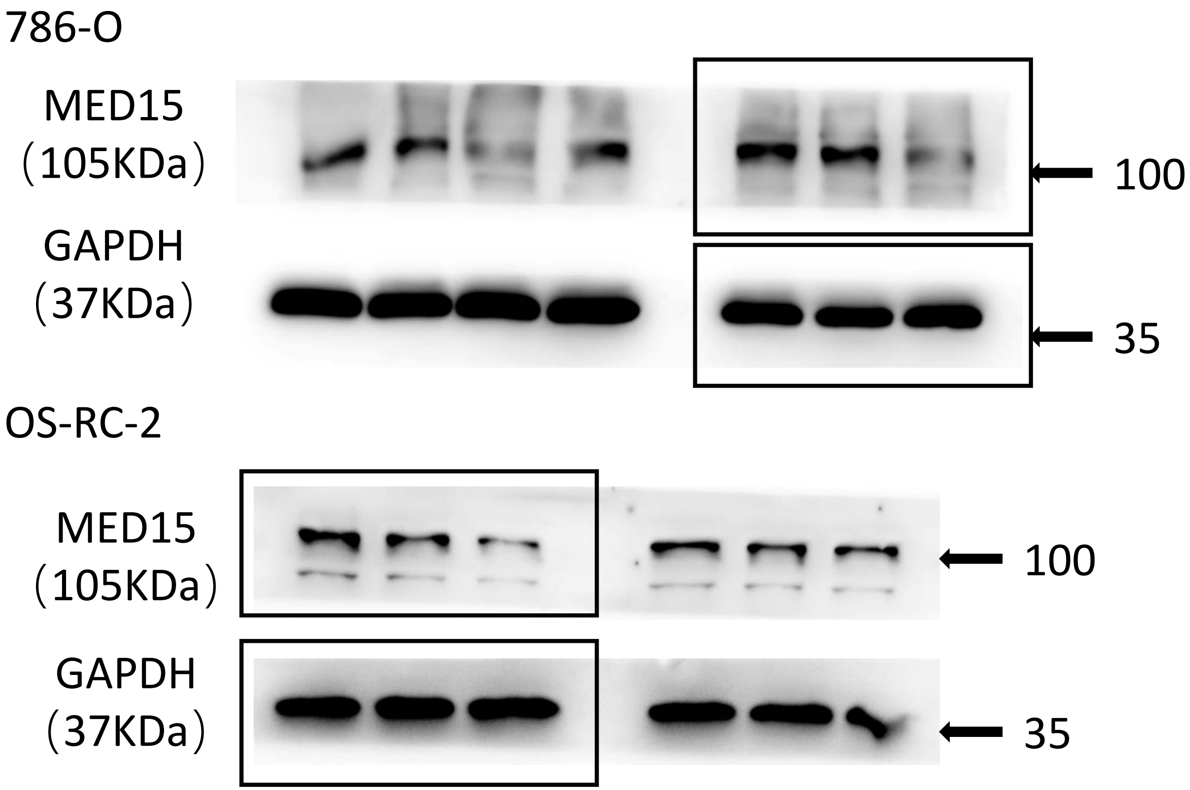
786-O
MED15
(105KDa)
GAPDH
(37KDa)
100
35
OS-RC-2
MED15
(105KDa)
GAPDH
(37KDa)
100
35

Figure 2H

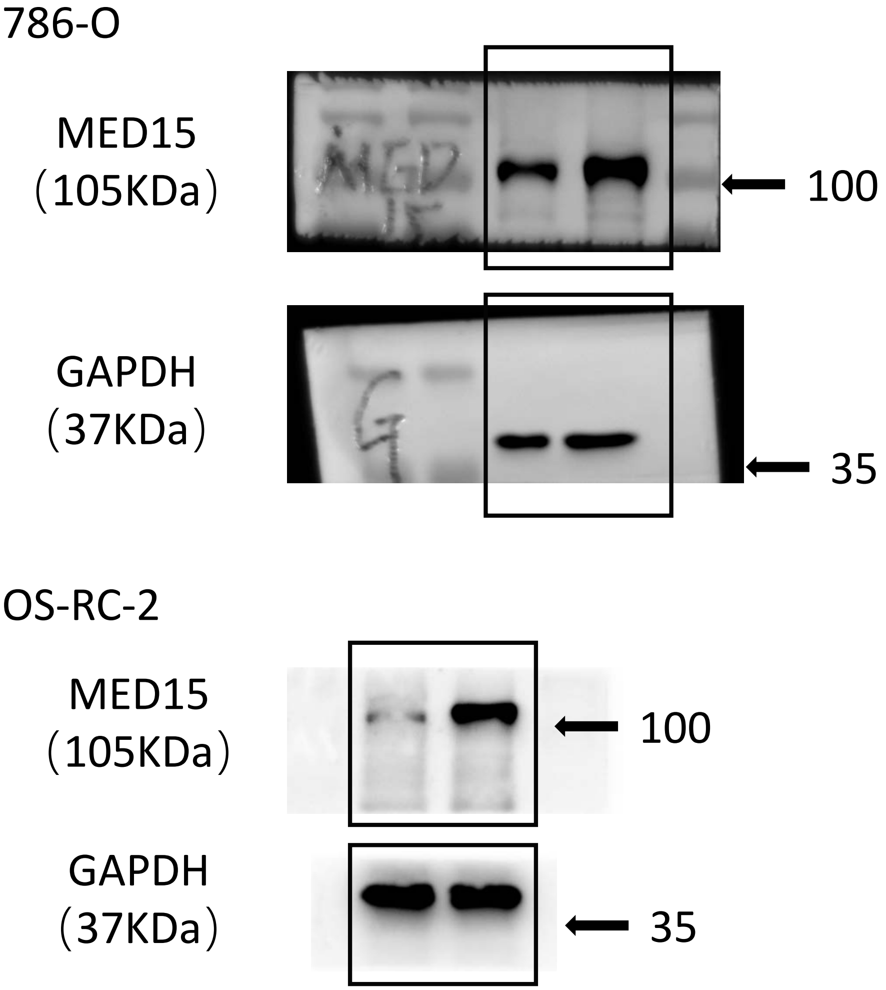

786-O
MED15
(105KDa)
100
GAPDH
(37KDa)
35
OS-RC-2
MED15
(105KDa)
100
GAPDH
(37KDa)
35

Figure 4

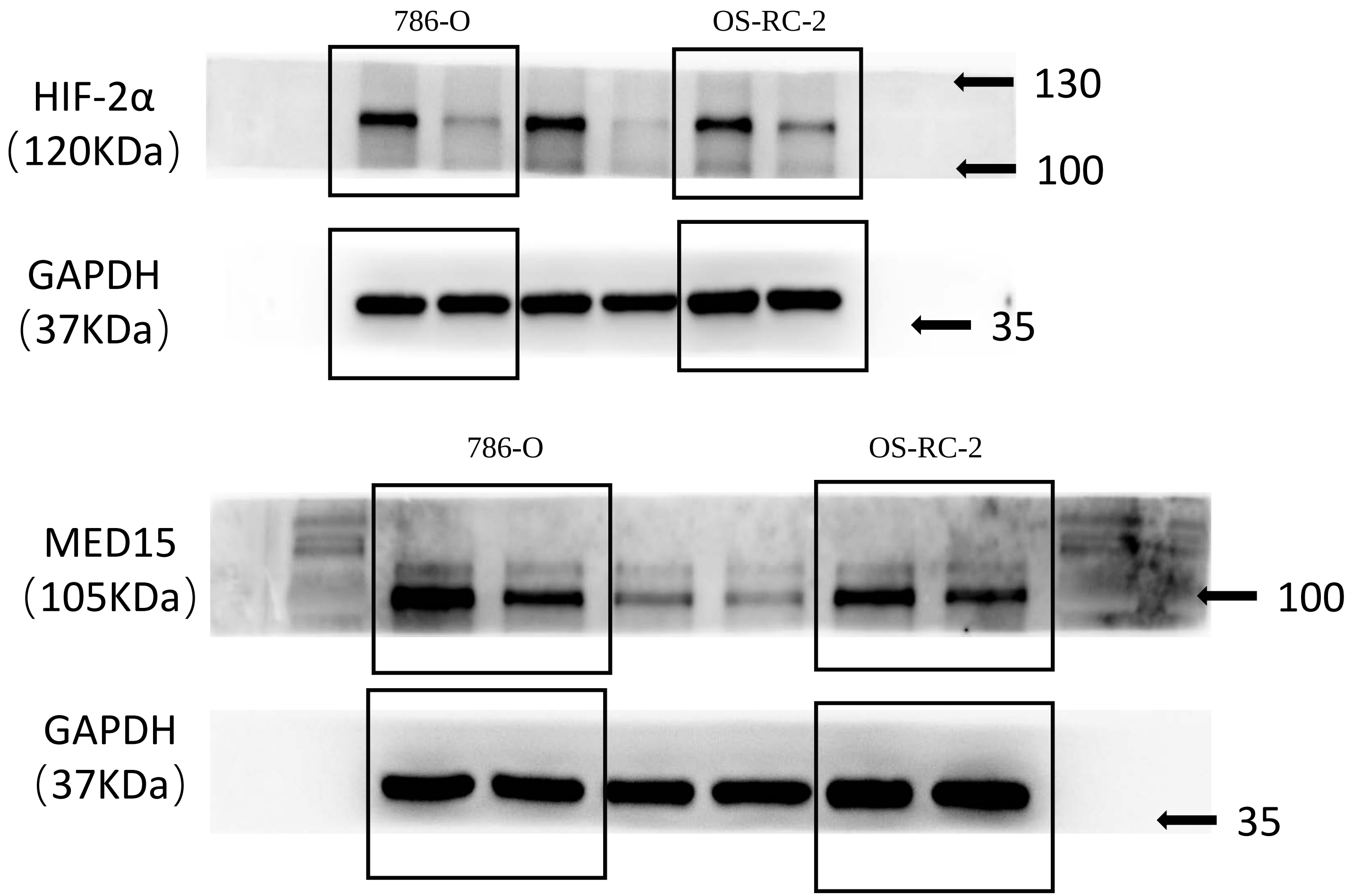
786-O
OS-RC-2
HIF-2α
(120KDa)
130
100
GAPDH
(37KDa)
35
786-O
OS-RC-2
MED15
(105KDa)
100
GAPDH
(37KDa)
35

Figure 5C

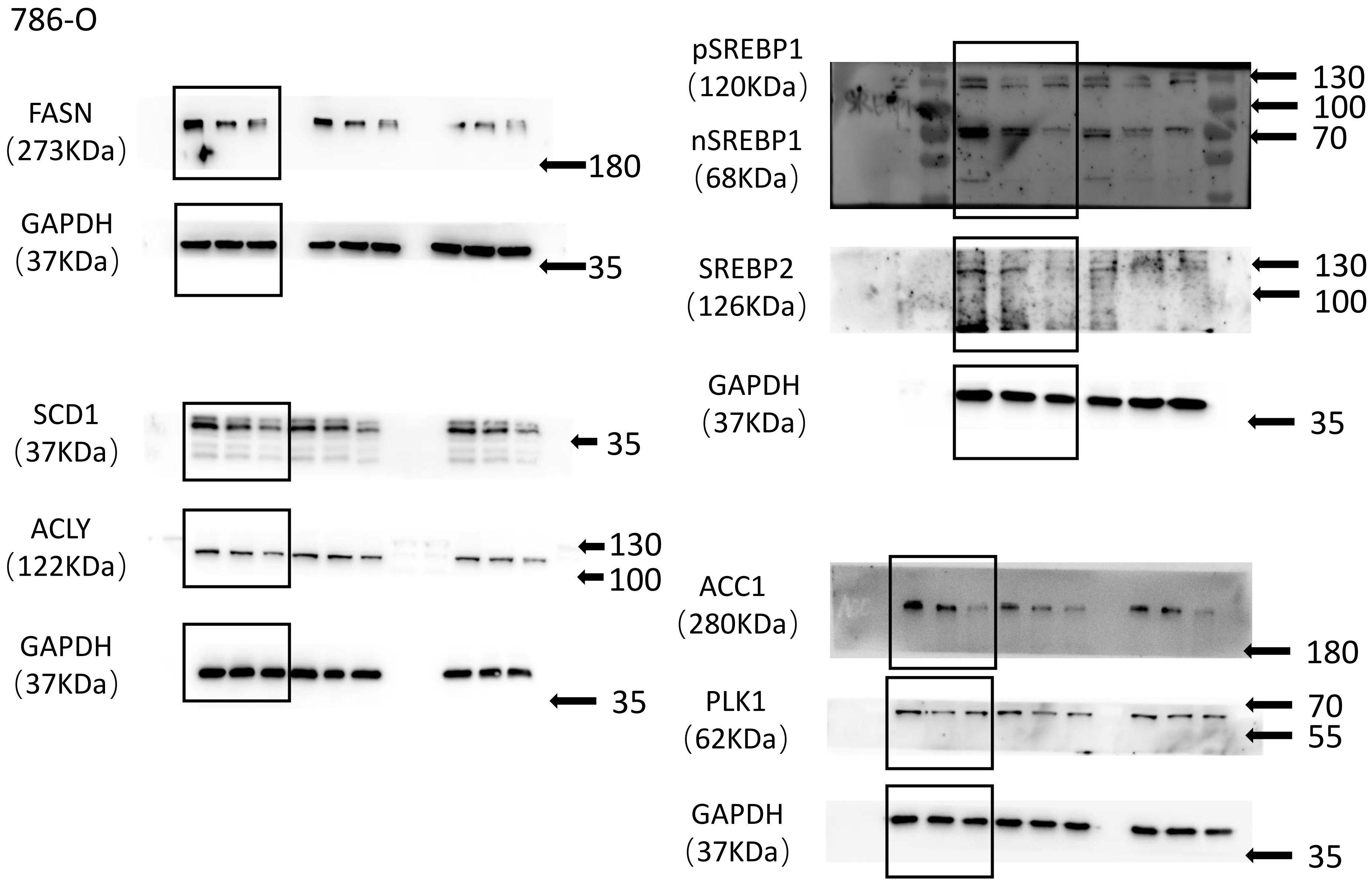
786-O
FASN
(273KDa)
180
GAPDH
(37KDa)
35
SCD1
(37KDa)
35
ACLY
(122KDa)
130
100
GAPDH
(37KDa)
35
pSREBP1
(120KDa)
nSREBP1
(68KDa)
130
100
70
SREBP2
(126KDa)
130
100
GAPDH
(37KDa)
35
ACC1
(280KDa)
180
PLK1
(62KDa)
70
55
GAPDH
(37KDa)
35

Figure 5C

OS-RC-2

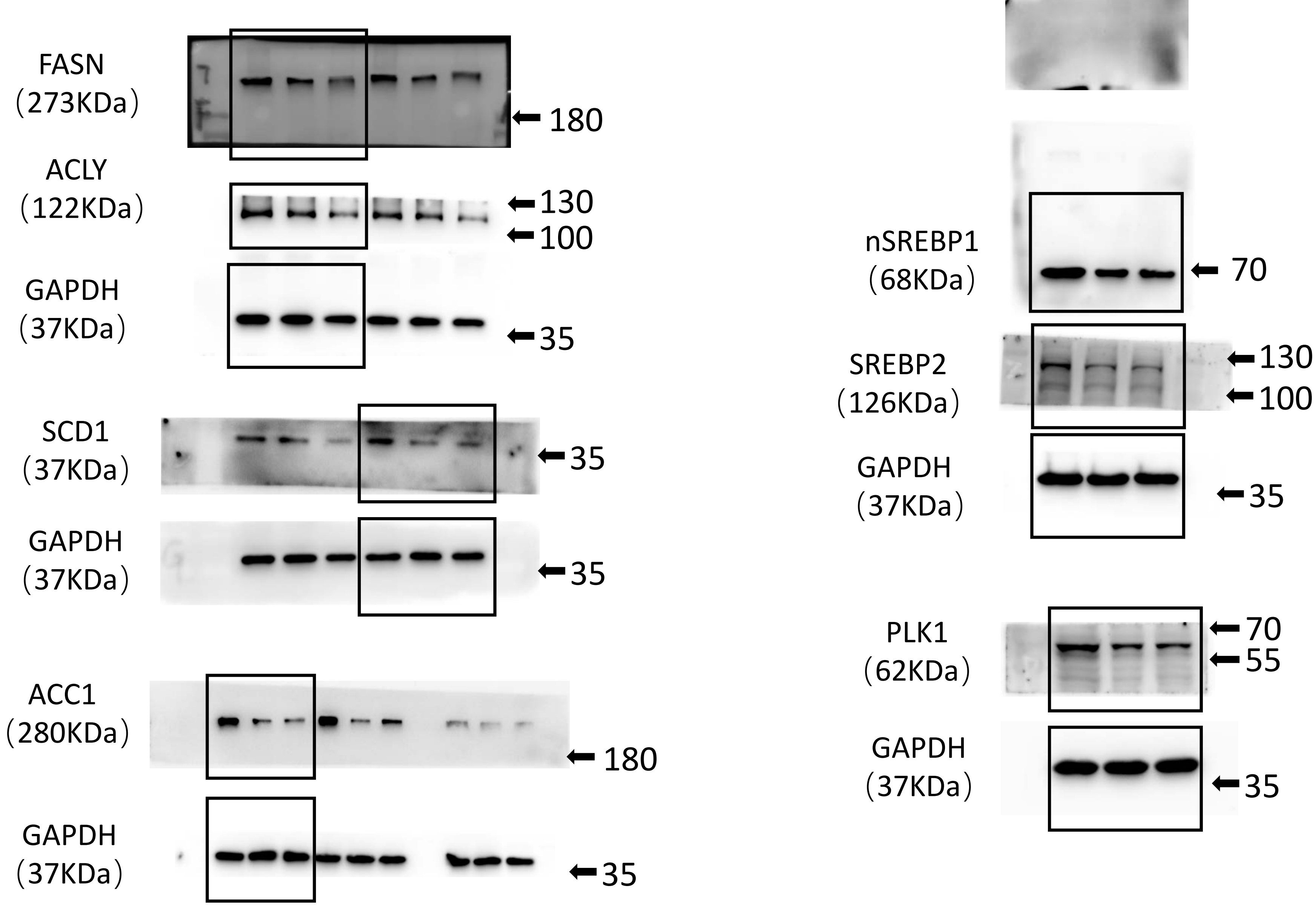

Figure 5E

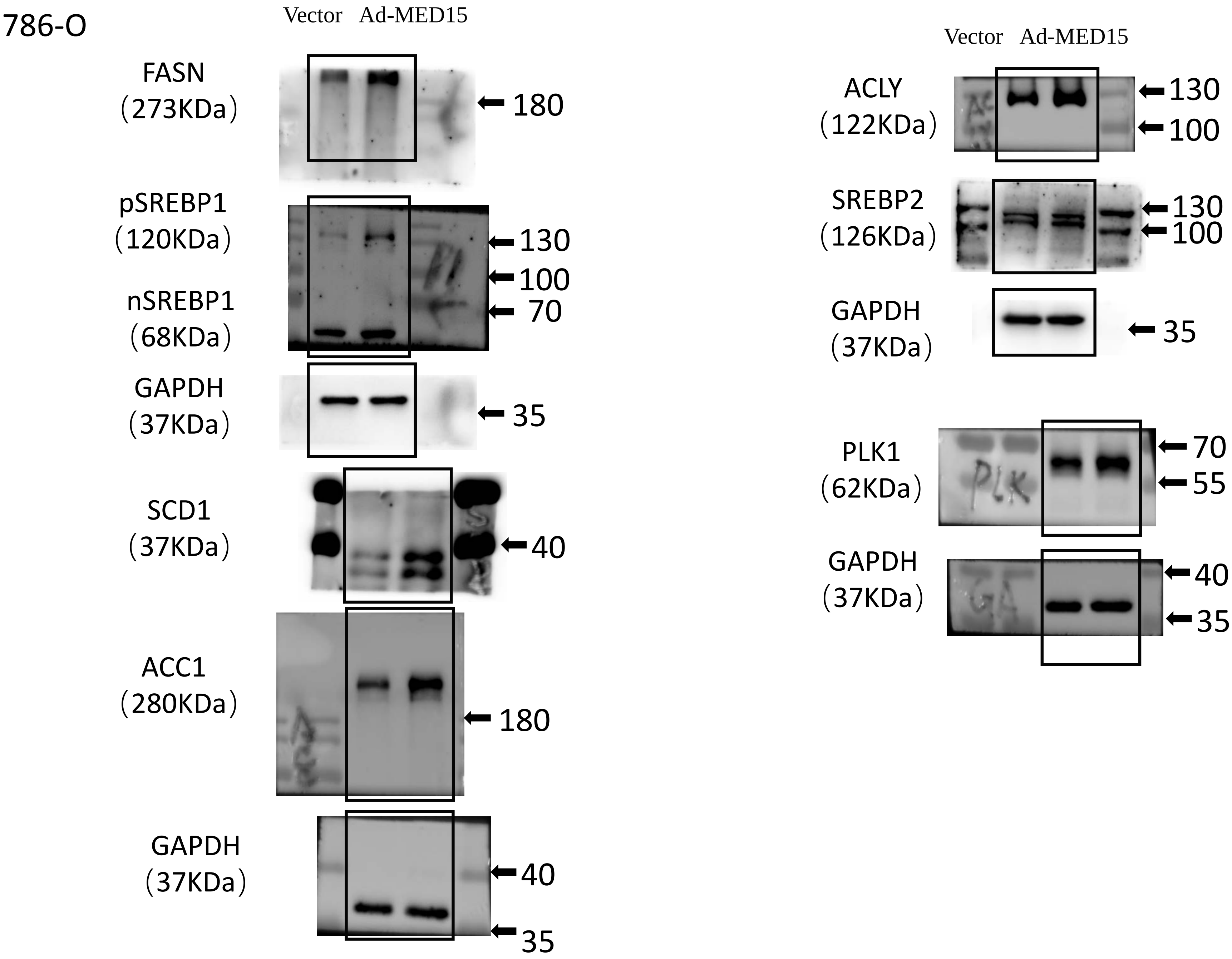
786-O
Vector Ad-MED15
FASN (273KDa)
180
pSREBP1 (120KDa)
nSREBP1 (68KDa)
130
100
70
GAPDH (37KDa)
35
SCD1 (37KDa)
40
ACC1 (280KDa)
180
GAPDH (37KDa)
40
35
Vector Ad-MED15
ACLY (122KDa)
130
100
SREBP2 (126KDa)
130
100
GAPDH (37KDa)
35
PLK1 (62KDa)
70
55
GAPDH (37KDa)
40
35

Figure 5E

OS-RC-2

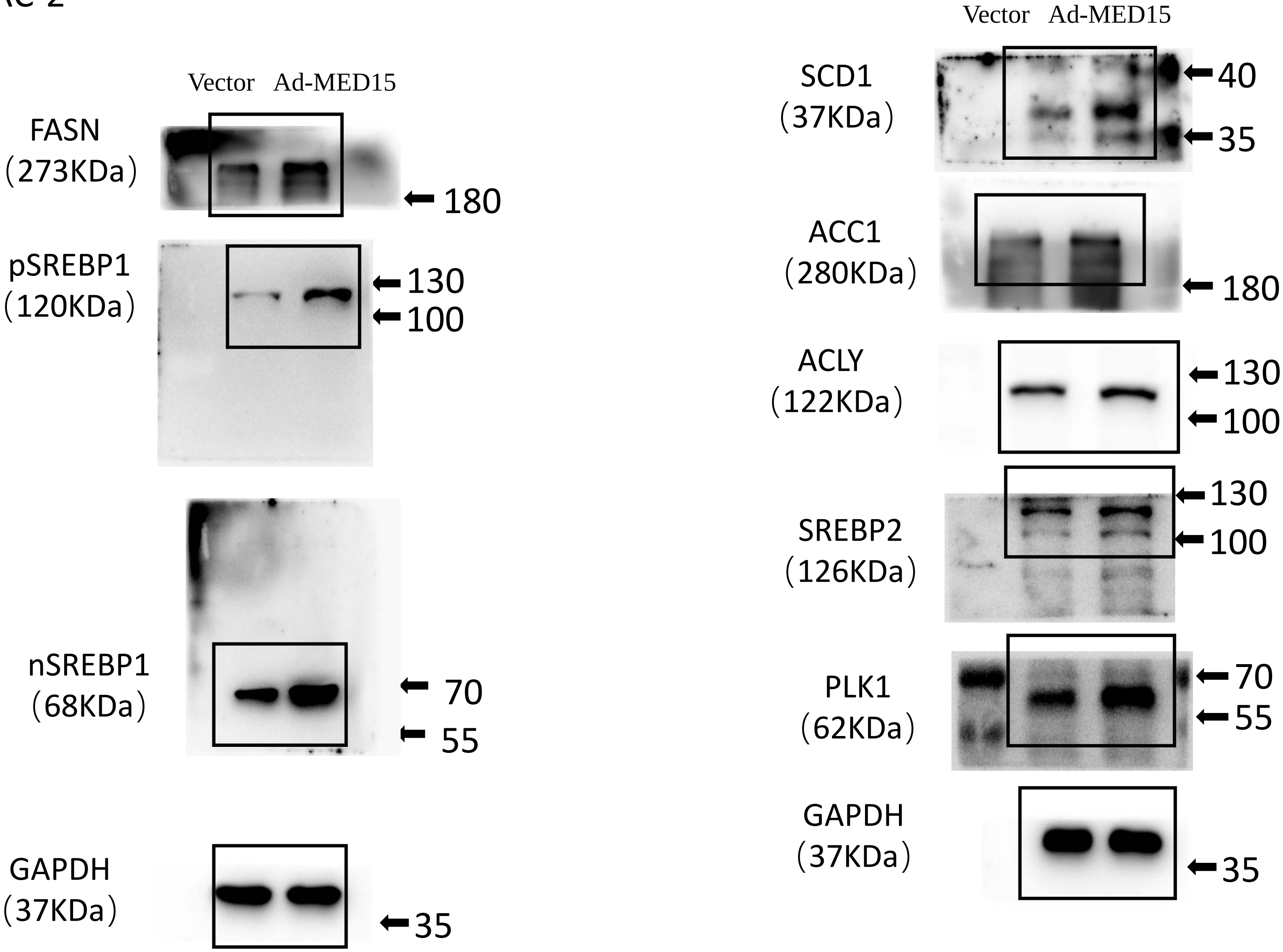

Figure 5G

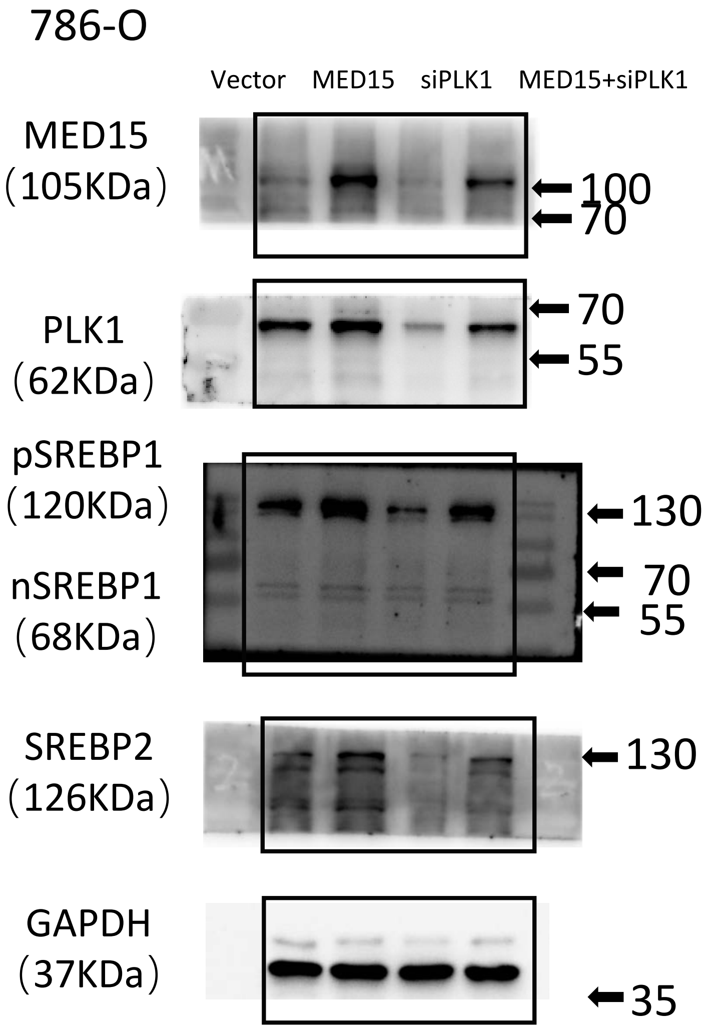

786-O
Vector
MED15
siPLK1
MED15+siPLK1
MED15
(105KDa)
100
70
PLK1
(62KDa)
70
55
pSREBP1
(120KDa)
130
nSREBP1
(68KDa)
70
55
SREBP2
(126KDa)
130
GAPDH
(37KDa)
35

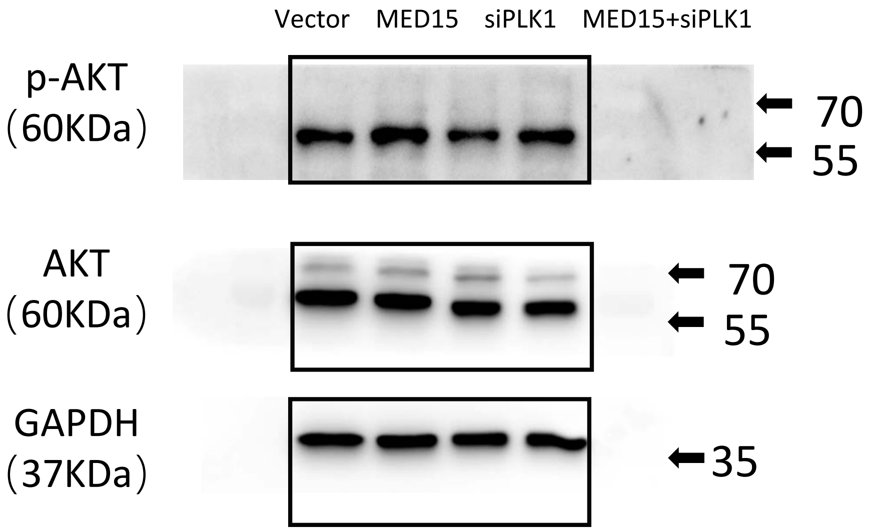

Vector
MED15
siPLK1
MED15+siPLK1
p-AKT
(60KDa)
70
55
AKT
(60KDa)
70
55
GAPDH
(37KDa)
35

Figure 5G

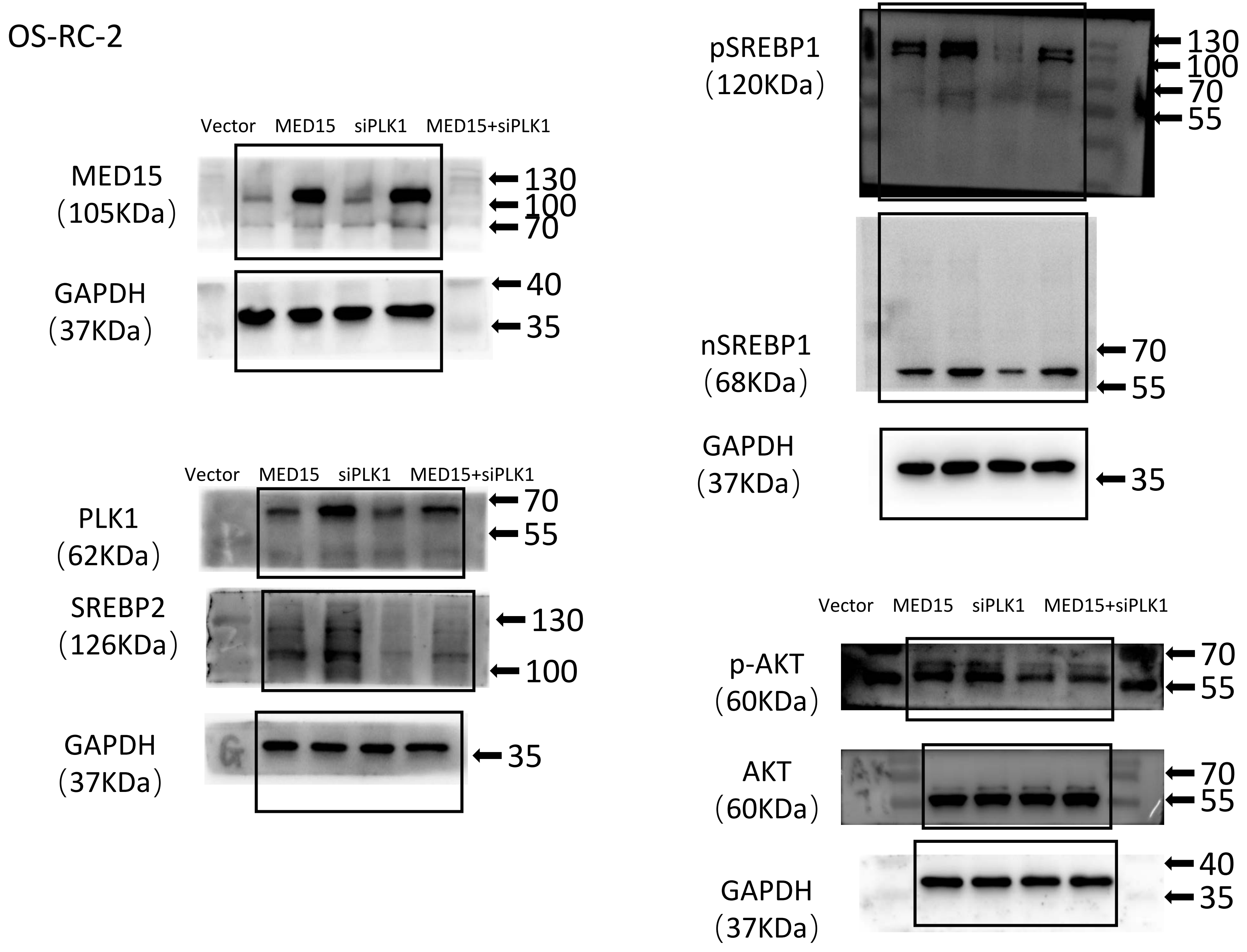
OS-RC-2
Vector
MED15
siPLK1
MED15+siPLK1
MED15
(105KDa)
GAPDH
(37KDa)
130
100
70
40
35
PLK1
(62KDa)
SREBP2
(126KDa)
GAPDH
(37KDa)
70
55
130
100
35
pSREBP1
(120KDa)
130
100
70
55
nSREBP1
(68KDa)
70
55
GAPDH
(37KDa)
35
p-AKT
(60KDa)
AKT
(60KDa)
GAPDH
(37KDa)
70
55
70
55
40
35

Figure 6A

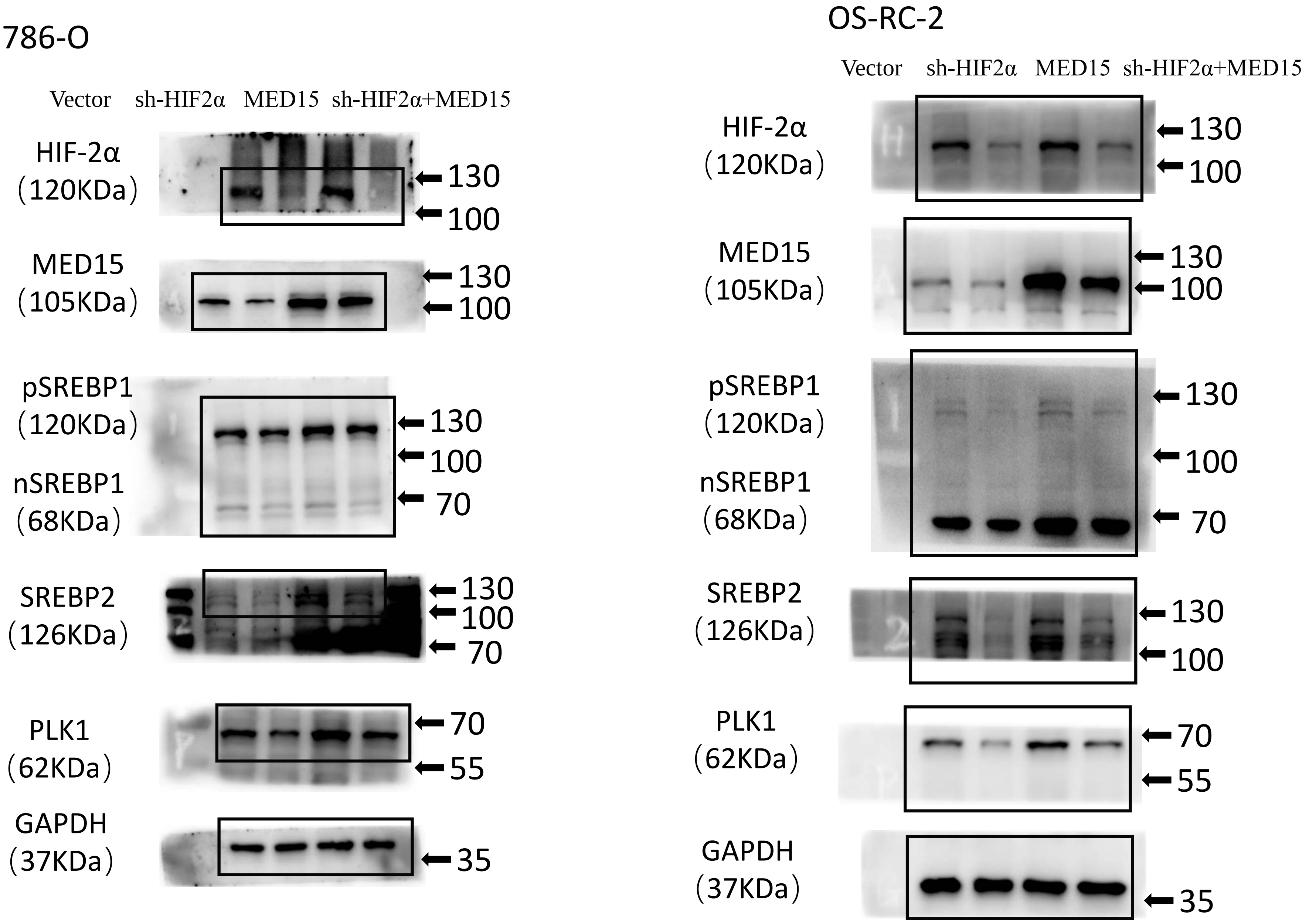

786-O
Vector
sh-HIF2α
MED15
sh-HIF2α+MED15
HIF-2α
(120KDa)
130
100
MED15
(105KDa)
130
100
pSREBP1
(120KDa)
nSREBP1
(68KDa)
130
100
70
SREBP2
(126KDa)
130
100
70
PLK1
(62KDa)
70
55
GAPDH
(37KDa)
35
OS-RC-2
Vector
sh-HIF2α
MED15
sh-HIF2α+MED15
HIF-2α
(120KDa)
130
100
MED15
(105KDa)
130
100
pSREBP1
(120KDa)
nSREBP1
(68KDa)
130
100
70
SREBP2
(126KDa)
130
100
PLK1
(62KDa)
70
55
GAPDH
(37KDa)
35
